# Cryo-EM reveals that *Escherichia coli* tRNA-transglycosylase can bind and act upon two tRNAs

**DOI:** 10.1073/pnas.2601895123

**Published:** 2026-07-21

**Authors:** Alexander Harjung, Ember M. Ruth, Mariusz Matyszewski, Jaehee Park, Caroline Knittel, Evan McCormack, Neal K. Devaraj

**Affiliations:** ^a^ Department of Chemistry and Biochemistry, University of California, San Diego, CA 92093; ^b^ Department of Molecular Biology, School of Biological Sciences, University of California, San Diego, CA 92093

**Keywords:** cryo-EM, posttranscriptional modification, RNA, transglycosylase, RNA binding protein

## Abstract

Bacterial tRNA-guanine transglycosylases (TGT) are essential enzymes involved in tRNA modification, contributing to the virulence of multiple pathogens. TGT from *Escherichia coli* was the first protein of this family to be isolated and purified, and as such has served as a model enzyme for the biochemical characterization of TGTs. *E. coli* TGT is also one of the most disease-relevant TGTs, sharing high sequence identity with TGTs from several human pathogenic bacteria, including *Shigella* spp. and *Salmonella* spp. Notably, TGTs from some *Shigella* strains are sequence-identical to the *E. coli* enzyme. In addition, as a highly promiscuous enzyme, *E. coli* TGT has found use as an RNA-modification tool in chemical biology, enabling site-specific covalent RNA modification in vitro and in vivo. For these reasons, there has been significant interest in solving the structure of *E. coli* TGT. However, crystallization of *E. coli* TGT has proven difficult, and to date, structural insights have relied on surrogate TGT enzymes from other organisms. Here, we present the cryo-EM structure of *E. coli* TGT and its covalent intermediate with a full-length tRNA. Unexpectedly, the structure reveals that the *E. coli* TGT dimer binds and acts upon two tRNAs, which is unlike all other known TGTs. Closer analysis of the TGT–tRNA complex reveals several important interactions outside of the enzyme’s active site, that facilitate RNA binding and stabilize the conformational change of the tRNA anticodon loop. Based on these structural insights, we were able to design improved, high-affinity, TGT substrate RNA hairpins.

## Significance

tRNA base-modification is an important mechanism for the regulation of protein expression on the RNA level. Several disease-causing bacteria use the enzyme tRNA-guanine transglycosylase (TGT) to upregulate translation of virulence factors which are essential for facilitating host infection. As such, TGT is a potential drug target for diseases like shigellosis. In this work, we present the cryo-EM structure of *Escherichia coli* TGT, identical to the *Shigella* enzyme, and investigate the structural basis of its interaction with tRNA. Our work reveals that, unlike all other known TGTs, the *E. coli* TGT homodimer forms covalent intermediates with two tRNAs. These findings advance our understanding of TGT enzymology and provide a structural foundation for the development of antibiotics and next-generation RNA-labeling tools.

The tRNA-guanine transglycosylases (TGTs) are key enzymes involved in the post-transcriptional modification of tRNAs (1). The biochemical activity of TGTs varies across the different domains of life. In eubacteria, TGT enzymes typically catalyze the insertion of preQ_1_ into tRNAs, whereas in eukaryotes, the enzymes directly incorporate queuine salvaged from the environment. In archaea, the enzymes modify tRNAs by inserting archaeosine instead (2). Despite these differences, TGTs are a highly conserved enzyme class across all three domains of life. In bacteria, TGT enzymes catalyze the exchange of the nucleobase guanine for preQ_1_ in the anti-codon loop (wobble position) of specific tRNAs. As a precursor to the highly modified nucleobase queuine, the insertion of preQ_1_ into tRNAs is thought to fine-tune protein expression at the translational level (3).

Due to its conserved role in translational regulation, bacterial TGT has been extensively studied as a potential drug target, particularly in the context of infectious diseases such as shigellosis (bacillary dysentery), caused by *Shigella* spp (4). This disease is responsible for an estimated 1.1 million deaths annually worldwide (5). Due to the recent emergence of antibiotic-resistant *Shigella* strains, there is a need for novel therapeutic options (6). It has been shown that the inhibition of TGT can downregulate the expression of several key virulence proteins of *Shigella* by interfering with translation of the transcription factor *virF*. This significantly reduces the pathogenicity of *Shigella* which makes TGT a promising drug target (7–9).

Apart from its role in bacterial infection, TGT has also found extensive application in chemical biology. *E. coli* TGT has been shown to be highly promiscuous and can insert a variety of substrates into tRNAs. Remarkably, TGT does not require the full tRNA (10) for activity: a shortened anticodon loop sequence, termed TAG3, is sufficient for TGT to catalyze nucleobase exchange. TGT can therefore be used to modify almost any RNA containing the TAG3 handle with a variety of covalent modifications (11). This site-specific RNA labeling technology (RNA-TAG) has been employed for a variety of applications like light activated CRISPR-gene editing in cells (12), controlling translation by modifying mRNA (13), site-specific labeling of RNA for cellular imaging (14, 15) as well as for increasing mRNA stability and translation efficiency in mice (16). In addition, apart from its natural RNA substrate, *E. coli* TGT is also able to modify DNA, further increasing the scope of its applications (17, 18).

Several structures of TGTs from different organisms have been solved, including those from *Zymomonas mobilis* (19), and human TGT (20–22). These structures have revealed important details about the architecture of the TGT active site, dimerization interface (23), as well as substrate and inhibitor interactions (24–27). In contrast, *E. coli* TGT has been challenging to crystallize, and consequently, its structure has remained unsolved (1).

While TGT active sites are highly conserved, sequence identity can vary highly even across different bacterial strains. So far, the structure of *Z. mobilis* TGT has been used in structure-guided drug design approaches. However, the sequence identity between *Z. mobilis* TGT and *Shigella* TGT is only 56% (1). *Shigella* TGT instead is nearly identical to *E. coli* TGT with sequence identities between 99% and 100% depending on the bacterial strain. *E. coli* TGT is also closely related to *Salmonella* spp. TGTs with sequence identities over 90% (28). Due to its important role in infectious disease and its application in chemical biology, solving the structure of *E. coli* TGT has been a longstanding goal in the field.

Bacterial TGTs typically exist as homodimers and previous reports have suggested higher-order oligomer formation. As such we hypothesized that, despite the enzyme’s small size (42 kDa), cryoelectron microscopy (cryo-EM) might be used for elucidating the structure of *E. coli* TGT. Here we present the structure of *E. coli* TGT solved by cryo-EM. We also investigate how *E. coli* TGT interacts with one of its cognate tRNAs, tRNA^Tyr^ by purifying the TGT–tRNA covalent intermediate complex and solving the structure using cryo-EM. Currently, the accepted mechanism of action for all known TGTs is that only one of the monomers in the functional TGT dimer is catalytically active, while the other monomer has an unoccupied active site and helps to orient and stabilize the single bound tRNA (29–32). Unexpectedly, we find that the functional *E. coli* TGT dimer binds and engages two tRNAs. Our study demonstrates that *E. coli* and *Shigella* TGTs likely differ significantly from *Z. mobilis* TGT in how they engage their substrate tRNAs to perform posttranscriptional modification. To the best of our knowledge, this is the first report of a TGT protein from any domain of life for which both monomers in the functional dimer can form a covalent intermediate with RNA. While previous structural studies have elucidated the interactions of tRNA with the highly conserved TGT active site, for *E. coli* TGT several additional amino acids outside of the TGT active site stabilize binding of the tRNA stem. By using mutational analysis, we confirm the importance of these interactions for formation of the covalent intermediate which is required for enzymatic activity. Based on these findings, we then designed bivalent, dual hairpin RNA substrates capable of engaging both active sites of the TGT dimer simultaneously. Using a competitive labeling assay against a conventional, single hairpin substrate, we show that RNA substrates capable of binding to both active sites at the same time show much higher labeling efficiency. As such, the identification of a unique dual mode of tRNA binding not only adds to our understanding of TGT biology but also facilitates structure-guided approaches for the design of high affinity TGT binding motifs and inhibitors.

## Results and Discussion

### Cryo-EM Structure of *E. coli* TGT.

*E. coli* TGT [99 to 100% sequence identity to *Shigella* spp. TGT, >90% sequence identity to *Salmonella* spp. TGT (28)] was expressed using a (T7)-2 vector (NEB) with a N-terminal strep-tag and a C-terminal His_6_-tag. Expression in BL21 (DE3)pLysS (Promega) and subsequent purification using affinity chromatography and size exclusion chromatography yielded highly active and pure TGT protein. The protein retained full activity without the need to remove purification tags and was therefore directly used for structural studies via cryo-EM.

**Fig. 1.**
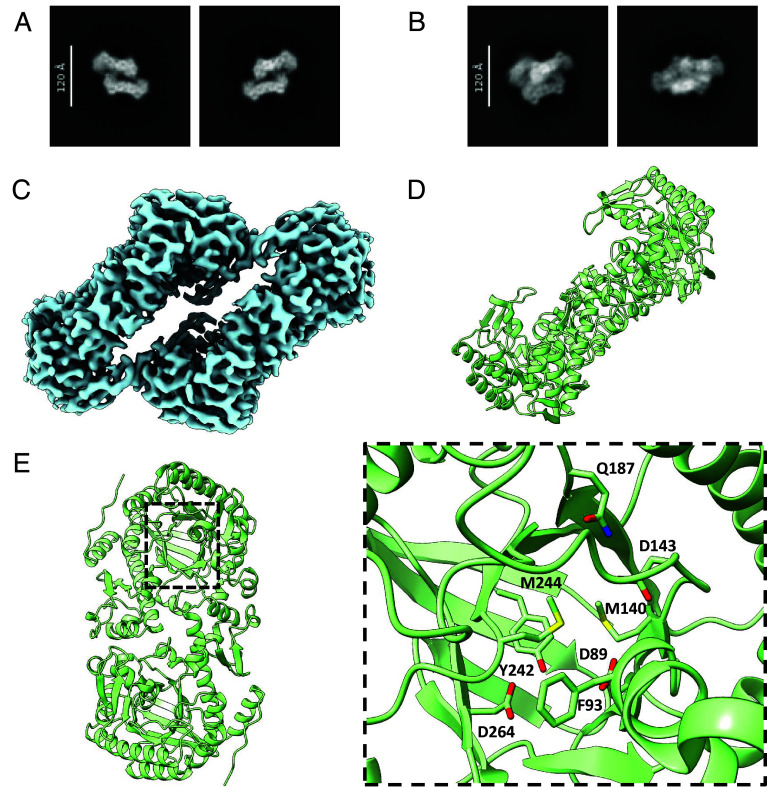
Cryo-EM structure of *E. coli* TGT. (*A*) 2D-class averages showing the TGT dimer of dimers particle that was used for solving the structure of TGT. (*B*) 2D-class averages showing TGT complexes of higher stoichiometry. (*C*) Side-view of the sharpened 3.5 Å resolution cryo-EM map of the *E. coli* TGT dimer of dimers particle. (*D*) Model of the functional TGT dimer built from the cryo-EM map. **(*E*)** Active site of *E. coli* TGT.

While in humans, TGT functions as a heterodimer made up from the two subunits QTRT1 and QTRT2 (21), in bacteria TGT has been shown to form a homodimer (19, 23). Additionally, for several TGTs, multimers of a higher stoichiometry have been reported. Accordingly, it has been hypothesized that *E. coli* TGT forms complexes of different stoichiometry in a concentration-dependent manner (33, 34). Initial cryo-EM experiments revealed that TGT indeed forms complexes of different stoichiometries depending on protein concentration. While at a protein concentration of 25 µM, *E. coli* TGT mostly forms homodimers (*SI Appendix*, Fig. S1), we could also observe some particles representing a dimer of a dimer. The TGT dimer particle exhibited a strong orientation bias and was therefore not suitable for solving the structure. By increasing protein concentration to 110 µM, dimer particles completely disappeared, and a majority of the particles adopted the tetrameric (dimer of dimers) conformation (Fig. 1*A* and *SI Appendix*, Fig. S2). Interestingly, in addition to the tetrameric conformation we could also occasionally observe complexes of higher stoichiometries, confirming past reports of higher-order *E. coli* TGT multimers. (Fig. 1*B*) (33, 34).

Using the dimer of dimers particles, we were able to solve the structure of *E. coli* TGT to a resolution of 3.5 Å (Fig. 1 *C* and *D*). As has been shown across TGTs from all domains of life, the enzyme’s active site is highly conserved. Accordingly, the active site of *E. coli* TGT closely resembles those of other TGT structures. All the residues implicated in the catalytic mechanism, including D264, the nucleophilic residue responsible for attacking the ribose backbone of substrate tRNAs, are also present in the *E. coli* TGT active site (Fig. 1*E*) (35). When compared to *Z. mobilis* TGT, the most notable difference is residue F93 in the active site. This position is occupied by a tyrosine in *Z. mobilis*. This tyrosine is thought to contribute to the catalytic cycle of the enzyme by acting as a hydrogen bond donor that interacts with the preQ_1_ substrate. However, since F93 is commonly found in eukaryotic TGTs such as those from humans and *C. elegans*, as well as in many other bacterial TGTs, this hydrogen bonding interaction does not appear to be essential for catalytic activity (25).

### The *E. coli* TGT Dimer Can Covalently Bind Two tRNAs.

As the natural substrates of TGT are its 4 cognate tRNAs (tRNA^Tyr^, tRNA^Asp^, tRNA^His^, tRNA^Asn^) we next set out to analyze how *E. coli* TGT binds tRNAs. As part of its mechanism of action, TGT forms a covalent intermediate through aspartate 264 in its active site with the ribose backbone of the anticodon loop of its substrate tRNA (1). It has been shown that through addition of 9-deazaguanine, the TGT reaction can be arrested at the covalent intermediate state, which makes isolation and purification of the covalent intermediate complex possible (36).

**Fig. 2.**
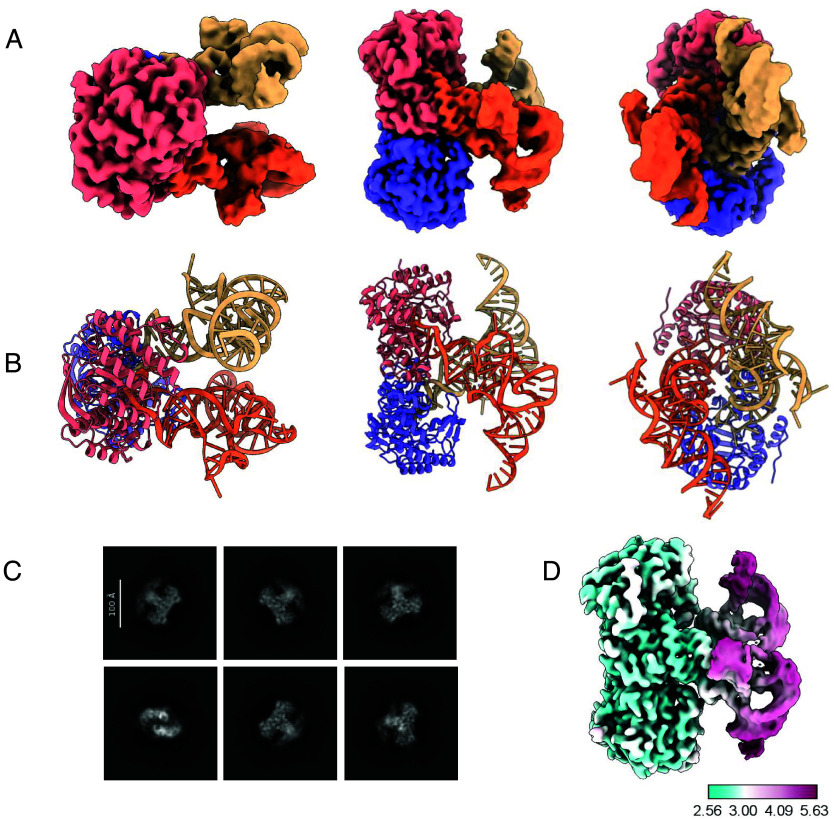
Cryo-EM structure of the *E. coli* TGT tRNA^Tyr^ covalent intermediate. (*A*) Representative views of the unsharpened cryo-EM map of the TGT–tRNA^Tyr^ covalent intermediate at a global resolution of 2.72 Å. Monomers of TGT in the functional homodimer are colored in blue and red. tRNA^Tyr^ covalently bound to the red TGT monomer is colored in orange. tRNA^Tyr^ covalently bound to the blue TGT monomer is colored in ochre (*B*) Corresponding model of the TGT–tRNA^Tyr^ covalent intermediate built from cryo-EM map. (*C*) Representative 2D-class averages of the TGT–tRNA^Tyr^ covalent intermediate clearly showing the presence of 2 tRNAs in the TGT dimer. (*D*) Local resolution map with resolution scale (Å).

After setting up a reaction between TGT and tRNA^Tyr^ in the presence of 9-deazaguanine, we confirmed formation of the covalent intermediate via SDS-PAGE and purified the resulting RNA–protein complex via size exclusion chromatography. Covalent intermediate containing fractions were pooled and the structure of the complex was solved using cryo-EM. The consensus in the literature for different TGTs across all domains of life is that in the functional TGT-homodimer [or heterodimer in the case of eukaryotes (21)], only one monomer is catalytically active and can bind RNA in its active site, while the other monomer is thought to orient the substrate RNA without being catalytically active itself or being able to accept an RNA substrate in its active site. Previous structural studies of bacterial TGTs (*Z. Mobilis*) have provided a rationale by suggesting that the binding of one tRNA to one active site of the TGT dimer sterically blocks the other active site from accepting another tRNA substrate (29–32). Unexpectedly, we found that the *E. coli* TGT–tRNA complex substantially differs from this canonical model. Initial cryo-EM experiments showed two distinct particles with either one or two tRNAs bound. While short incubation time of the covalent intermediate reaction mixture led to TGT particles with either one or two tRNAs bound, longer incubation time (6 h on ice) gave a homogenous sample containing only the TGT particle with two tRNAs bound. To solve a structure for both single and double bound TGT–tRNA covalent intermediates from the same sample, we limited the reaction time to one hour on ice. These conditions are similar to those used for the previously published covalent intermediate structure of *Z. mobilis* TGT, which was formed over 1 h at 25 °C (36). From the resulting 3D reconstructions, we can deduce that the *E. coli* TGT dimer appears to be able to bind and form a covalent intermediate with 2 tRNAs, where each tRNA occupies the active site of one monomer in the functional TGT dimer. The complex shows C2-symmetry, with the two tRNAs being oriented away from each other to avoid steric interference (Fig. 2 *A* and *B*). While the final cryo-EM map was generated with assigned C2-symmetry, we were also able to show the presence of 2 tRNAs using C1 ab initio reconstruction and C1 nonuniform refinement (*SI Appendix*, Fig. S3). Similarly, 2D classification (Fig. 2*C*) also clearly confirms two tRNAs bound to the TGT-dimer. The cryo-EM maps show strong density for the ligand 9-deazaguanine (9DG) in the TGT active sites. In addition, clear density for the covalent linkage between D264 and ribose 35 of the tRNA is observed in the reconstruction (*SI Appendix*, Fig. S4). Apart from the active site, the tRNAs also seem to interact with other regions of TGT which likely helps stabilize and orient the tRNA in the active site. While these regions of the tRNAs were resolved to high resolution, more peripheral regions of the tRNAs proved to be surprisingly flexible and subsequently resolved to lower resolution (Fig. 2*D*). Accordingly, modeling these distal RNA regions was more challenging, and their interpretation is limited to placement of the RNA backbone trace rather than precise base or side-chain conformations. Similar to other known TGT–tRNA covalent intermediate structures (36, 37), the tRNA anticodon loop adopts an unusual conformation in which the loop is flipped up almost 180° to fully access the enzyme’s active site.

Due to orientation bias, the structure of the covalent intermediate with only one tRNA bound was resolved to lower resolution (*SI Appendix*, Fig. S5). Nevertheless, our results clearly indicate that the *E. coli* TGT dimer can bind either one or two tRNAs in its active site. We therefore hypothesize that single and double bound states can exist in equilibrium.

### Binding Interactions Between tRNA and TGT Outside of the Active Site Are Required for Formation of the Covalent Intermediate.

Previous work on *Z. mobilis* TGT provided detailed insight into how the enzyme engages RNA within its active site (36). Because the active site is highly conserved among TGTs, the same residues mediate RNA interactions in the *E. coli* TGT active site. The above study also identified several additional binding interactions outside of the TGT active site. While these residues are conserved in *E. coli* and likely contribute to tRNA binding, we noticed several additional binding interactions that seem to be unique to *E. coli* TGT (*SI Appendix*, Fig. S6) and might help explain the difference in observed RNA binding of the two enzymes. Since most of these residues are not conserved in *Z. mobilis*, we set out to examine their contribution to tRNA binding and covalent intermediate formation.

**Fig. 3.**
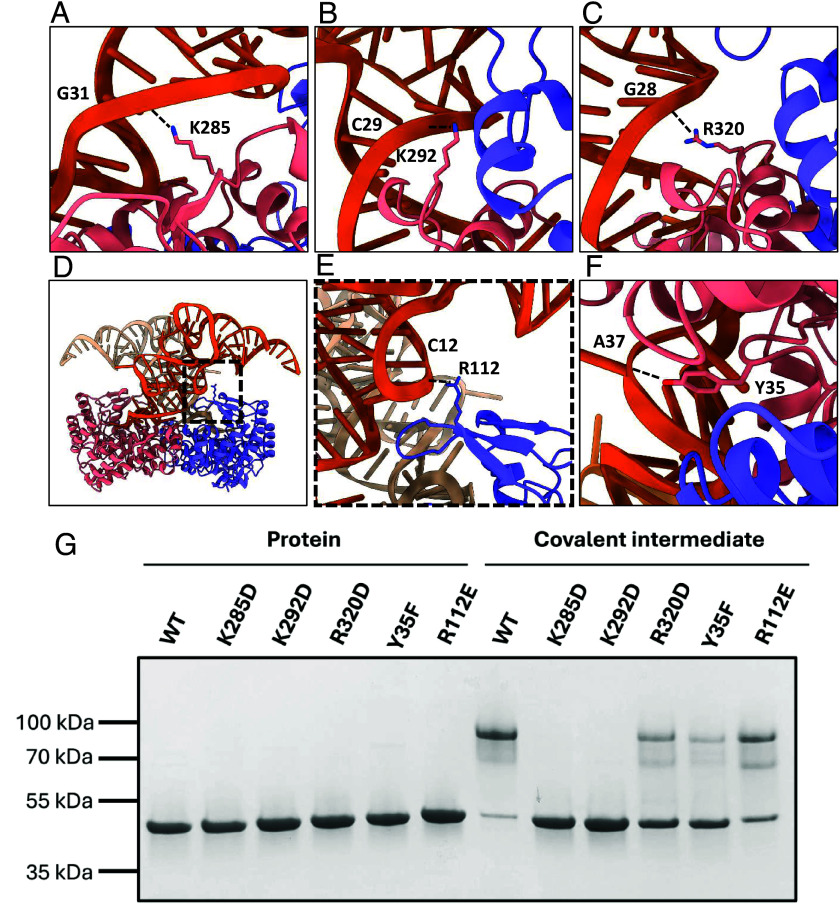
Several residues outside of the TGT active site stabilize the binding of tRNA^Tyr^. (*A–C*) Positively charged amino acids outside of the TGT active site that show strong interaction with tRNA^Tyr^. (*D* and *E*) R112 from one TGT monomer (blue monomer) stabilizes the tRNA (orange) covalently bound to the active site of the other TGT-monomer (red monomer). (*F*) Y35 stabilizing binding of the tRNA close to the active site. (*G*) Mutational analysis of TGT: SDS-PAGE gel comparing WT-TGT with 5 mutants (K285D, K292D, R320D, Y35F, R112E). For covalent intermediate formation, TGT (25 µM), tRNA^Tyr^ (37.5 μM), and 9-deazaguanine (500 μM) were incubated on ice for 6 h. Shown are the purified proteins and the proteins after covalent intermediate formation. TGT shows a single band migrating between 35 and 55 kDa, whereas the covalent intermediate migrates between 70 and 100 kDa. Some mutant TGT covalent intermediates show a faint second band of unclear origin, migrating at approximately 70 kDa. Experiments were replicated twice with comparable results.

Outside the active site, tRNA^Tyr^ shows interactions with several residues of *E. coli* TGT. In particular, a stretch of positively charged residues, K285, K292, and R320 appears to play a key role in stabilizing the binding of the tRNA stem (Fig. 3 *A–C*). Mutational analysis revealed that substitution of any of these residues with the negatively charged amino acid aspartate results in a severe loss of enzymatic function (Fig. 3*G*). While the R320D mutant (Fig. 3*C*), retains a limited capacity to form the covalent intermediate, its activity is substantially reduced compared to wild-type TGT. The K292D variant (Fig. 3*B*), by contrast, exhibits a complete loss of detectable activity. The same is true for K285D (Fig. 3*A*). The fact that these single point mutations completely disrupt the enzyme’s ability to form the covalent intermediate further highlights the importance of binding interactions of the tRNA with TGT outside of the active site.

As outlined above, structural studies of other TGTs (*Z. mobilis* and human) have shown that only one monomer of the TGT dimer forms a covalent intermediate with tRNA, while the second, noncatalytic monomer is proposed to stabilize and orient the bound tRNA. In contrast, this work demonstrates that *E. coli* TGT accommodates two tRNAs simultaneously, raising the question of whether comparable cross-monomer interactions occur. Our structural data indicate that, unlike in *Z. mobilis*, such interactions only play a minor role in *E. coli* TGT. Only R112 appears to stabilize the tRNA covalently linked to the opposite monomer’s active site, likely through a weak interaction with two phosphate oxygens of the tRNA^Tyr^ backbone at position C12 (Fig. 3 *D* and *E*). Interestingly, mutation of R112 to glutamate only slightly impairs the enzyme’s ability to form the covalent intermediate (Fig. 3*G*). We therefore propose that, in *E. coli* TGT, intermolecular stabilization of tRNA across the two monomers plays a secondary role and might not even be necessary at all for enzymatic function. This further highlights the difference between *E. coli* TGT–tRNA binding and other well-studied TGTs, in which cross-monomer RNA binding and stabilization within the dimer are thought to be critical (4, 29).

Comparison of the RNA interacting residues described above across various TGTs (*SI Appendix*, Fig. S7) reveals that they are conserved in some species but highly divergent in others. For instance, many disease-relevant bacteria (*Shigella* spp., *Salmonella* spp., *V. cholerae*) share the exact same RNA binding region. While R112 and K292 are functionally conserved in *Z. mobilis* (as K and R, respectively), K285 is not.

Close to the enzyme’s active site, the tRNA anti-codon loop shows a strong conformational change compared to free tRNA. This conformational change is characteristic for TGT–tRNA covalent intermediates (36, 37) and entails an upward flip of the loop of almost 180° at A37 of the tRNA. To accommodate this unusual conformational change of the tRNA backbone, three bases, U34, A37, and A39, are flipped out of the tRNA. Tyrosine Y35 (Fig. 3*F*) interacts with the RNA-backbone at A37, likely stabilizing the unusual tRNA fold. Indeed, mutational analysis revealed that a Y35F mutation leads to significantly decreased formation of the covalent intermediate (Fig. 3*G*). We therefore hypothesize that Y35 acts as a hydrogen bond donor, engaging the RNA-backbone.

In contrast, in *Z. mobilis* TGT, Y35 is mutated to alanine and therefore cannot participate in RNA binding as a hydrogen bond donor (*SI Appendix*, Fig. S7).

As the above-described mutations change the electrostatic and hydrogen bonding profile of the tRNA binding interface, likely weakening the binding interaction, they offer a possible explanation for the difference in tRNA binding between the two homologous bacterial enzymes. These findings suggest the existence of distinct RNA-binding mechanisms even among closely related bacterial TGTs.

### A Dynamic Loop (L98 to I104) Directly Participates in tRNA Recognition by Stabilizing the Characteristic tRNA Anticodon Loop Kink in the Covalent Intermediate.

**Fig. 4.**
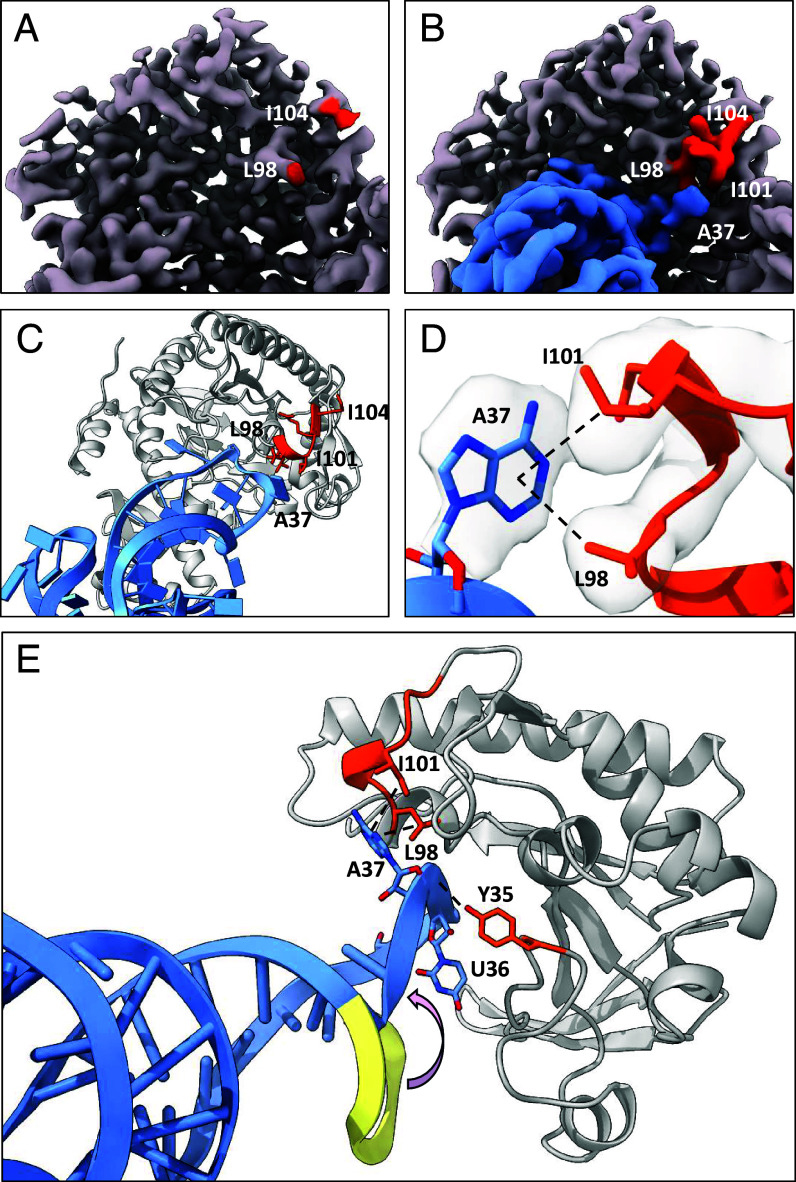
A dynamic loop (L98 to I104) directly participates in tRNA recognition by stabilizing the characteristic tRNA anticodon loop kink in the covalent intermediate. (*A*) Cryo-EM map of TGT: In the absence of tRNA, L98-104 is highly disordered showing only weak density. (*B*) Cryo-EM map of the TGT–tRNA covalent intermediate: In the tRNA (blue) bound state, TGT (gray) shows strong density for L98-I104 (orange). (*C* and *D*) A flipped-out base (A37) stacks against L98 and I101 in the covalent intermediate structure. (*E*) tRNA^Tyr^ (blue) undergoes a conformational change upon TGT binding. tRNA^Tyr^ is highlighted in blue and the expected orientation of the anticodon loop of a free tRNA is shown in yellow (based on the structure of tRNA^Phe^, PDB 1TRA). The anticodon loop is flipped upward by almost 180˚ compared to a free tRNA. The flipped-up anticodon loop is stabilized by stacking of A37 against L98 and I101 as well as by the interaction between Y35 and the RNA backbone.

Apart from sterically accommodating the conformational change of the tRNA, base flipping also facilitates tRNA binding to TGT by enabling direct interactions between protein side chains and the flipped-out nucleotide. Comparison of the electron density maps of TGT and the covalent TGT–tRNA intermediate reveals a pronounced difference in density for a short loop encompassing residues L98 to I104 (Fig. 4 *A* and *B*). In the absence of tRNA, this region lacks interpretable density, indicative of substantial conformational flexibility (Fig. 4*A*). In contrast, in the tRNA-bound complex this loop becomes well-ordered and is resolved with strong density (Fig. 4*B*), consistent with ligand-induced stabilization. Interestingly, this ordering seems to be mediated through interaction with one of the flipped-out RNA bases: A37 stacks against residues L98 and I101 at distances characteristic of hydrophobic base–side-chain interactions (Fig. 4 *C* and *D*). The emergence of well-defined density for this loop specifically upon tRNA binding, together with its direct engagement of the flipped base, strongly suggests that these interactions contribute to stabilizing the protein–tRNA complex. Notably, the base-stacking interactions between A37 and residues L98 and I101 are positioned in immediate proximity to the pronounced kink in the tRNA backbone (Fig. 4*E*). A37 is located at the center of the flipped-up loop, such that stabilization of this base by hydrophobic stacking necessarily constrains the local RNA geometry. The close spatial coupling between base stabilization and backbone distortion suggests that interactions between the above-described loop and the flipped-out base, as well as Y35’s interaction with the RNA backbone, may contribute not only to tRNA binding but also to stabilizing the kinked, catalytically relevant tRNA conformation. Consistent with this model, the ordering of the loop encompassing residues L98 to I104 is observed exclusively in the tRNA-bound complex, supporting a role for these interactions in maintaining the conformationally strained RNA geometry required for catalysis.

### Structure Based Design of High Affinity, Dual TAG3_2_ Hairpins for RNA-TAG.

RNA modifications are emerging as powerful biotechnological tools for tuning the performance of synthetic and therapeutic RNAs. Base modifications such as N^1^-methylpseudouridine or m^7^G cap analogs can dramatically improve mRNA stability, translation efficiency, and innate immune evasion, which is why almost all mRNA therapeutics use modified nucleotides. While the addition of unnatural or modified nucleotides during RNA-transcription enables the synthesis of highly modified mRNAs, this process lacks control and leads to random incorporation of modified nucleotides across the whole mRNA. Site-specific modification strategies provide a solution to this problem by making it possible to place a given modification only where it is mechanistically useful.

**Fig. 5.**
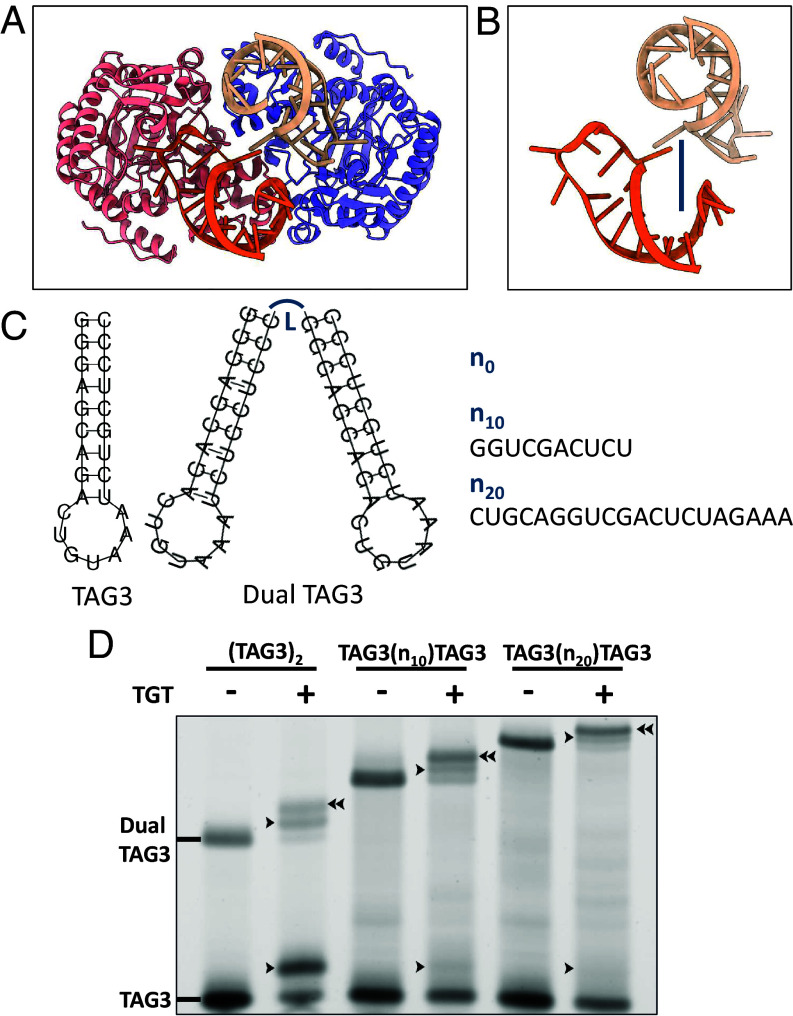
Structure-based design of dual TAG3_2_ TGT substrates with increased binding affinity. (*A*) Model of the TGT–tRNA^Tyr^ covalent intermediate with only the TAG3 RNA residues shown. (*B*) Linking the two TAG3 hairpins forms a TAG3 dimer with higher binding affinity to TGT than TAG3 alone. (*C*) TAG3 constructs with different linker lengths (n_0_, n_10_, n_20_) were designed and analyzed in a competitive labeling assay with conventional single TAG3. (*D*) Urea-page gel analysis of a competitive TGT labeling assay. Labeling of TAG3 constructs with preQ_1_-biotin was used to compare the labeling efficiency of TAG3 and dual TAG3_2_ constructs (10 µM single TAG3, 2.5 µM dual TAG3_2_, 1.25 µM TGT; 37 °C, 4 h). Based on the gel-shift indicating preQ1-biotin insertion, the new dual TAG3_2_ constructs are more efficient substrates for TGT than the single TAG3. Despite the large excess of TAG3 (10 µM) over the dual TAG3_2_ constructs (2.5 µM), dual TAG3_2_ constructs outcompete TAG3. Constructs with n_10_ and n_20_ linkers show that increased linker length improves binding affinity. The single arrow indicates single labeling (labeling of one hairpin per TAG3 construct). Double arrows indicate double labeling (labeling of both hairpins in one dual TAG3_2_ construct).

Due to its ability to post-transcriptionally and selectively modify RNA, TGT has become a valuable tool for site-specific RNA modification. Its utility stems from its ability to recognize a short RNA hairpin motif (TAG), originally derived from the anticodon loop of tRNA^Tyr^, and incorporate modified nucleobases at defined positions. These recognition sequences can be added to virtually any RNA of interest, enabling site-specific labeling or functionalization simply by inserting the TAG recognition motif upstream or downstream of the target sequence (11–16, 18). Building on this technology and utilizing our recent structural insights described above, we explored ways to improve the efficiency of TGT-mediated RNA labeling. Specifically, since we were able to show that *E. coli* TGT forms a homodimer capable of simultaneously engaging the anticodon loop of two tRNAs, we hypothesized that designing a bivalent RNA substrate that can simultaneously occupy both active sites of the TGT-dimer could increase binding affinity and catalytic efficiency. To test this idea, we designed a series of RNA constructs that contain two tandem TAG hairpins. Since we knew of the important binding interactions between TGT and its RNA substrate outside of the active site, we decided on using a binding motif that mimics the tRNA^Tyr^ stem-loop and is long enough to interact with all the amino acids that we observed to facilitate tRNA-binding (TAG3 hairpin, Fig. 5 *A* and *B*). Based on our structural data we assumed that simply connecting two TAG3 hairpins could give an RNA that can bind both active sites of the TGT dimer. However, we also wanted to investigate how adding an additional spacer sequence between the two TAG3 hairpins influences reaction efficiency. We hypothesized that adding a flexible linker between the two TAG3 hairpins might lead to better simultaneous binding to both TGT active sites, as well as help establish binding interactions between the enzyme and the tRNA. We therefore designed “dual TAG3_2_ hairpin” constructs that were connected either directly or via short nucleotide linkers of defined lengths (Fig. 5*C*). We then evaluated these constructs in an RNA-TAG labeling assay (11) using a preQ_1_-biotin probe to biotinylate the RNA-constructs as a test reaction.

To determine if dual TAG3_2_ are better substrates for TGT labeling compared to single TAG3, we performed a competitive labeling assay. By having a single TAG3 compete with dual TAG3_2_ in the same labeling reaction, any difference in enzyme–substrate engagement will strongly influence the final degree of labeling of each respective hairpin. Due to their difference in size, single and dual TAG3_2_ hairpins migrate differently on urea-page gels, which enabled us to directly compare the reaction efficiency of our dual hairpin RNAs to the standard single TAG3 motif. Incorporation of the preQ_1_-biotin probe into our RNA substrates could be confirmed via gel shift analysis due to the increase in molecular weight of the final product. To challenge the potential high binding affinity of our dual TAG3_2_ constructs, we included an excess of single TAG3 (10 µM single TAG3, 2.5 µM dual TAG3_2_ construct) and 1.25 µM of TGT per reaction.

As is shown in Fig. 5*D*, dual TAG3_2_ hairpins are significantly better substrates for TGT labeling compared to single TAG3 hairpins. For dual TAG3_2_ (n_0_), we observed some labeling of single TAG3. However, for both constructs with linkers (n_10_ and n_20_), single TAG3 was completely outcompeted by dual TAG3_2_ and no single TAG3 labeling could be observed despite it being present at a 4× excess in each reaction. All dual TAG3_2_ RNAs readily undergo double labeling, with the n_20_ construct appearing to be completely double labeled after 4 h at 37 °C. Double labeling is not necessarily a result of both TGT active sites acting on one hairpin simultaneously, but could also be a result of independent labeling or even binding events. Based on our data, the n_20_ dual TAG3_2_ construct is the best substrate out of all the substrates we tested. We hypothesize that the longer linker enables the dual TAG3_2_ constructs to better engage both active sites of the TGT dimer, leading to higher binding affinity. The inability of the TAG3_2_ (n0) construct to outcompete single TAG3 further highlights the advantage conferred by the ability to simultaneously bind both active sites of the TGT dimer. Like the other dual TAG3_2_ constructs, n0 contains two modifiable hairpins and would be expected to benefit from an avidity effect, whereby binding of one hairpin positions the second in close proximity to the active site of the opposing TGT monomer. However, unlike the n10 and n20 constructs, n0 does not fully outcompete single TAG3. We hypothesize that this reflects weaker effective binding to both active sites, likely due to reduced flexibility and/or unfavorable geometric constraints. This observation supports the conclusion that the n10 and n20 constructs are capable of simultaneously binding to both active sites of the TGT dimer, resulting in higher overall binding affinity.

To further investigate the hypothesized higher binding affinity of dual TAG3_2_ constructs relative to single TAG3, we evaluated the binding affinities of TAG3 and the best-performing dual TAG3_2_ construct (n20) using differential scanning fluorimetry (DSF), monitoring shifts in protein melting temperature (T_m_) as a function of ligand concentration (*SI Appendix*, Fig. S8). In this assay, ligand binding is inferred from stabilization of the protein fold, reflected as an increase in T_m_ with increasing RNA concentration. Increasing concentrations of TAG3 or TAG3(n_20_)TAG3 were mixed with TGT and melting temperatures (T_m_) were determined using the fluorescent dye SYPRO™ Orange. Both RNAs induced concentration-dependent stabilization of the protein, however, the dual TAG3_2_ construct produced a substantially larger thermal shift and reached saturation at lower concentrations. Fitting the T_m_ values to a single-site binding model yielded an apparent K_D_ in the low micromolar range (~3 to 5 µM) for TAG3, whereas the dual TAG3_2_ construct exhibited significantly tighter binding with an apparent K_D_ of ~0.7 to 1 µM. In addition to the enhanced affinity, the dual-construct produced a markedly greater maximal stabilization, consistent with stabilizing effects arising from simultaneous engagement of both binding sites by a single RNA.

## Conclusion

In this work we present a cryo-EM structure for *E. coli* TGT. Due to the high sequence identity, between *E. coli* TGT and TGTs from several human pathogenic bacteria, including *Shigella* and *Salmonella*, the presented structures have relevance beyond *E. coli*. We were able to show that *E. coli* TGT can adopt complexes of different stoichiometry depending on protein concentration. We further investigated how *E. coli* TGT interacts with one of its cognate tRNAs, tRNA^Tyr^. By stabilizing the covalent intermediate with 9-deazaguanine, we were able to isolate and characterize a TGT–tRNA^Tyr^ complex by cryo-EM. Solving the structure of the covalent intermediate formed between TGT and *E. coli* tRNA^Tyr^ revealed an unexpected mode of substrate binding by the *E. coli* TGT dimer, challenging the prevailing model in which only one monomer within the functional dimer is catalytically active and capable of binding tRNA. Surprisingly, the structure shows that both monomers of the *E. coli* TGT dimer can simultaneously engage substrate tRNAs, forming covalent intermediates at each active site. The resulting complex displays C2 symmetry, with each tRNA occupying a distinct active site.

This dual engagement suggests a revised understanding of TGT function in *E. coli* and *Shigella*, where both subunits may be catalytically active. In addition to active site interactions, we observed tRNA contacts with peripheral regions of the protein, which likely contribute to substrate positioning and stabilization. The anticodon loop of each tRNA undergoes conformational changes that facilitate active site accommodation, consistent with previously solved TGT–tRNA-covalent intermediate structures. Further analysis revealed that this conformation is stabilized by a stacking interaction between a flipped-out RNA-base and a disordered region of TGT that becomes ordered upon tRNA binding.

We also observed a subpopulation of complexes containing a single tRNA bound to the TGT dimer, suggesting that both singly and doubly bound states can exist in equilibrium. While it is not known how much TGT is expressed in *E. coli* and *Shigella* cells, we believe it is a reasonable estimate that the natural intracellular TGT concentration is much lower than intracellular tRNA concentrations. The concentration of tRNA^Tyr^ alone for instance, is estimated to be in the low micromolar range depending on which growth phase *E. coli* cells are in (38). This means that tRNA^Tyr^ is likely to be present at a large excess compared to TGT. As such, we estimate that formation of covalent intermediate with two tRNAs bound to the TGT dimer can occur under physiological conditions.

We anticipate that our work also has important implications for the use of TGT as a chemical biology tool. Based on our finding that the *E. coli* TGT dimer can bind and act upon two tRNAs, we were able to design dual-TAG3_2_ hairpins, which can bind to both active sites of the TGT dimer, leading to higher binding affinities. In a competitive labeling assay, these dual-TAG3_2_ hairpins outcompeted single TAG3, with the labeling reaction going to completion for dual-TAG3_2_ while single TAG3 remained completely unlabeled. As such, the dual-TAG3_2_ hairpins present a useful addition to the TGT-toolbox, especially for applications where high binding affinities at low TGT and RNA concentrations are essential.

## Materials and Methods

### General Information.

tRNA^Tyr^ and all TAG3 RNAs were purchased from Cisterna biologics. HisPur™ Ni-NTA Resin was purchased from ThermoFisher (Cat# 88221). BL21(DE3)pLysS cells were purchased from Promega (Cat# L1195). All size exclusion chromatography was done using an ӒKTA pure™ 25 system. 9-deazaguanine was purchased from Cayman Chemicals (Cat# 21674).

### Oligonucleotides.

tRNA^Tyr^: GGUGGGGUUCCCGAGCGGCCAAAGGGAGCAGACUGUAAAUCUGCCGUCACAGACUUCGAAGGUUCGAAUCCUUCCCCCACCACCA

TAG3 [also known as ECYMH (33)]: GGGAGCAGACUGUAAAUCUGCUCCC

TAG3(n_0_)TAG3: GGGAGCAGACUGUAAAUCUGCUCCCGGGAGCAGACUGUAAA UCUGCUCCC

TAG3(n_10_)TAG3: GGGAGCAGACUGUAAAUCUGCUCCCGGUCGACUCUGGGAGCA GACUGUAAAUCUGCUCCC

TAG3(n_20_)TAG3: GGGAGCAGACUGUAAAUCUGCUCCCCUGCAGGUCGACUCUAG AAAGGGAGCAGACUGUAAAUCUGCUCCC.

### Protein Expression and Purification.

A sequence encoding TGT (Uniprot ID P0A847; The whole plasmid sequence can be found in the *SI appendix*) was cloned into the (T7)-2 vector (NEB). *E. coli* TGT (42,594 Da) was expressed with an N-terminal Strep-tag and a C-terminal His_6_-tag. The affinity tags were not removed prior to activity assays or structural characterization by cryo-EM. Proteins were expressed in BL21(DE3)pLysS. After transformation, a single colony was picked and used to grow a starter culture overnight at 37 °C. 20 mL of starter culture was added to 1 L LB with carbenicillin (100 µg/mL). The culture was grown on a shaker at 37 °C until it reached an OD of 0.6. After cooling to 18 °C, protein expression was induced through addition of 0.5 mM IPTG. The culture was incubated overnight at 18 °C. The next day, cells were harvested by centrifugation at 3,000×*g*.

Cells were lysed using sonication in 10 mL lysis buffer (20 mM Tris pH = 8.0; 500 mM NaCl; cOmplete™ protease inhibitor (Roche); 30 mM imidazole). The resulting cell lysate was clarified through centrifugation at 12,000×*g* for 20 min. Clarified lysate was purified on a gravity NiNTA column (1 mL of HisPur™ Ni-NTA resin) that had been equilibrated with lysis buffer. After incubation on an overhead spinner at 4 °C for 1 h, the lysate was passed through the column once. The column was washed 6 times with 1 mL of lysis buffer. Afterward, TGT was eluted from the column in 2.5 mL elution buffer (20 mM Tris pH = 8.0; 500 mM NaCl; 300 mM imidazole). The resulting protein was further purified using size-exclusion chromatography on a Superdex™ 75 Increase 10/300 GL column (Cytiva). Protein containing fractions were analyzed by SDS-PAGE. Fractions containing pure TGT were pooled and concentrated using an Amicon® Ultra Centrifugal Filter (10 kDa cutoff). For preparation of cryo-EM samples, TGT was concentrated to 8 mg/mL and octyl glucoside was added to a final concentration of 0.2% (w/v).

### Covalent Intermediate Formation for Cryo-EM.

A 500 μL TGT reaction was set up as follows: TGT (25 µM), tRNA^Tyr^ (37.5 μM), 9-deazaguanine (500 μM) were mixed in TGT reaction buffer (100 mM HEPES pH = 7.3, 5 mM DTT, 20 mM MgCl_2_). The reaction mixture (500 μL total volume) was incubated on ice for 60 min. Afterward, the covalent intermediate was purified on a Superose™ 6 Increase 10/300 GL column (Buffer: 20 mM Tris pH = 8.0, 300 mM NaCl). Fractions containing the covalent intermediate were pooled, concentrated to 1 mg/ml and used immediately for the preparation of cryo-EM samples.

### Mutational Analysis.

TGT mutants were generated using site directed mutagenesis (NEB). The primers used to generate each construct are listed in the Supplementary Information. All proteins were expressed and purified as described above for WT-TGT. A 10 μL TGT reaction was set up as follows for each TGT mutant: TGT (25 µM), tRNA^Tyr^ (37.5 μM), 9-deazaguanine (500 μM) were mixed in TGT reaction buffer (100 mM HEPES pH = 7.3, 5 mM DTT, 20 mM MgCl_2_). The reaction mixture (10 μL total volume) was incubated on ice for 6 h. 3.0 µL of each reaction were analyzed on SDS-PAGE.

### Electron Microscopy Sample Preparation and Data Collection.

All samples were prepared on UltraAuFoil R1.2/1.3, 300 mesh grids (Quantifoil) that had been freshly plasma-cleaned using a Gatan Solarus II plasma cleaner (10 s, 50 W, 75% Ar/25% O_2_ atmosphere), deposited with 3.0 μL of the protein solution, blotted for 4 sec, and then plunged into liquid ethane using a Vitrobot (Mark IV, Thermo Fisher Scientific). For the TGT structure, images were acquired on a Talos Arctica (FEI) operated at 200 kV equipped with a Falcon 4i Direct Electron Detector (Thermo Fisher) and collected using EPU with a total dose of 55 e/Å2 at 0.95 Å/pixel and a –2 to –1 µm defocus range.

For TGT–tRNA^Tyr^ covalent intermediate structures, images were acquired on a Titan Krios G4 (Thermo Fisher) operated at 300 kV and equipped with a Selectris X energy filter and a Falcon 4 Direct Electron Detector. Micrographs were collected using EPU with a total dose of 55 e/Å2 at 0.735 Å/pixel and at –2.2 to –0.6 µm nominal defocus range.

### Electron Microscopy Data Processing.

#### TGT structure.

Exposures were analyzed in cryoSPARC (39, 40). Initial particle picks were obtained using cryoSPARC Live’s blob picker (100 to 200 Å circular blobs), which were used to generate templates for one round of template picking. The resulting particles were cleaned up using one round of 2D-classification. After generating initial ab-initio models, the particle stack was further cleaned up by two rounds of heterogeneous refinement. The final reconstruction was done from 89,837 particles, with C2 symmetry assigned (41). The cryo-EM data processing workflow for the TGT structure is shown in more detail in *SI Appendix*, Fig. S9.

#### TGT–tRNA^Tyr^ covalent intermediate structures.

Exposures were analyzed in cryoSPARC (39, 40). Initial particle picks were obtained using cryoSPARC Live’s blob picker (100 to 200 Å circular blobs), which were used to generate templates for one round of template picking. The resulting particles were cleaned up using one round of 2D-classification. Initial 3D volumes were generated using multiclass ab-initio. The resulting volumes were used to further clean up the particle stacks through two rounds of 3D-classification. After one final round of heterogenous classification, the final structure was refined from 83,087 particles for TGT with two tRNAs bound and 45,089 particles for TGT with one tRNA bound. The cryo-EM data processing workflow for the TGT-covalent intermediate structures is shown in more detail in *SI Appendix*, Figs. S10 and S11.

#### Model building and refinement.

Initial models for model building were generated using AlphaFold (AlphaFold Server). AlphaFold models were fitted into cryo-EM maps and used for model building in Coot. Models were refined over several iterations using Phenix real space refine and manual refinement in Coot. The quality of the final models was assessed in Phenix. Figures containing cryo-EM maps or models were generated using ChimeraX.

#### Competitive labeling assay.

TGT reactions of all TAG3 RNA substrates were set up in the same manner with the following conditions: 10 µM TAG3, 2.5 µM TAG3_2_, 1.25 µM TGT; 20 μM PreQ1-biotin; 2 U RNAse inhibitor in TGT reaction buffer (100 mM HEPES pH = 7.3, 5 mM DTT, 20 mM MgCl_2_). The reaction components were combined and gently mixed by pipetting. Reactions were incubated for 4 h at 37˚C in a thermocycler with a heated lid. Afterward, reactions were directly analyzed via Urea PAGE.

#### Urea PAGE analysis.

For urea PAGE analysis of the single vs dual TAG3 competition reactions, the National Diagnostics 19:1 Acrylamide:Bisacrylamide Sequagel® UreaGel System (Catalog# EC-833) and the Mini-PROTEAN Tetra Vertical Electrophoresis Cell from Bio- Rad was used. Glass plates with 1.5 mm spacers were assembled using the Bio-Rad gel casting system. A 10 mL solution of 15% acrylamide was prepared in a 15 mL conical tube. TEMED (14 µL) was added, followed by 80 μL of 10% ammonium persulfate. The solution was mixed by inverting and poured between the assembled plates. A comb was inserted, and the gel was allowed to solidify for 20 min. Reaction samples were prepared using 2X RNA loading dye (NEB Catalog# B0363S), denatured at 98˚C for 10 min, loaded on the gel, and run at 100 V for 5 min, followed by 200 V for 60 min. Gels were removed and stained with GelRed (1:1,000 dilution; Biotium Catalog# 41011) in TBE for 20 min before imaging on a BioRad ChemiDoc.

#### DSF (42, 43).

TGT (2.5 µM) was mixed with SYPRO™ Orange (5× final concentration) in TGT reaction buffer (100 mM HEPES pH 7.3, 5 mM DTT, 20 mM MgCl_2_) on ice. Increasing concentrations of RNA (TAG3 or TAG3(n20)TAG3) were then added. The resulting mixtures were immediately subjected to melt curve analysis using SYPRO™ Orange fluorescence as a readout. Melt curves were generated in triplicate on a Bio-Rad CFX Real-Time PCR Detection System. Melt peak analysis was performed using the first derivative of the fluorescence signal (–dRFU/dT). Melting temperatures (T_m_) of TGT were plotted against the respective RNA concentration, and curves were fitted using a single-site binding model. The apparent K_D_ was inferred from the RNA concentration at the midpoint of the ΔT_m_ transition.

## Supplementary Material

Appendix 01 (PDF)

## Data Availability

The structures of *E. coli* TGT and its covalent intermediates with tRNA have been deposited into the Electron Microscopy Data Bank (EMDB) and the Protein Data Bank (PDB) with the following accession codes: *E. coli* TGT PDB 10FA (44)/EMD-75124 (45); *E. coli* TGT covalent intermediate with 1 tRNA PDB 10FB (46)/EMD-75125 (47); *E. coli* TGT covalent intermediate with 2 tRNAs PDB 10FC (48)/EMD-75126 (49).
