# Supplementary material for: Cryo-EM reveals that Escherichia coli tRNA-transglycosylase can bind and act upon two tRNAs
Source: Proc Natl Acad Sci U S A. 2026 Jul 21;123(30):e2601895123. doi: 10.1073/pnas.2601895123 (PMC13416341; doi:10.1073/pnas.2601895123)
Supplement: Supplementary file 1 — Appendix 01 (PDF) [file pnas.2601895123.sapp.pdf]

PNAS

Supporting Information for

Cryo-EM reveals that *E. coli* tRNA-transglycosylase can bind and act upon two tRNAs

Alexander Harjung,[1] Ember M. Ruth,[1] Mariusz Matyszewski,[2] Jaehee Park,[1] Caroline Knittel,[1] Evan McCormack,[1] Neal K. Devaraj[1]
1 Department of Chemistry and Biochemistry, University of California, San Diego, CA, USA
2 Cryo-EM facility, University of California, San Diego, CA, USA
*Neal K. Devaraj

This PDF file includes:

Figures S1 to S11
Tables S1 to S2
SI References

Fig. S1. Cryo-EM micrograph of *E. coli* TGT at 25 µM.

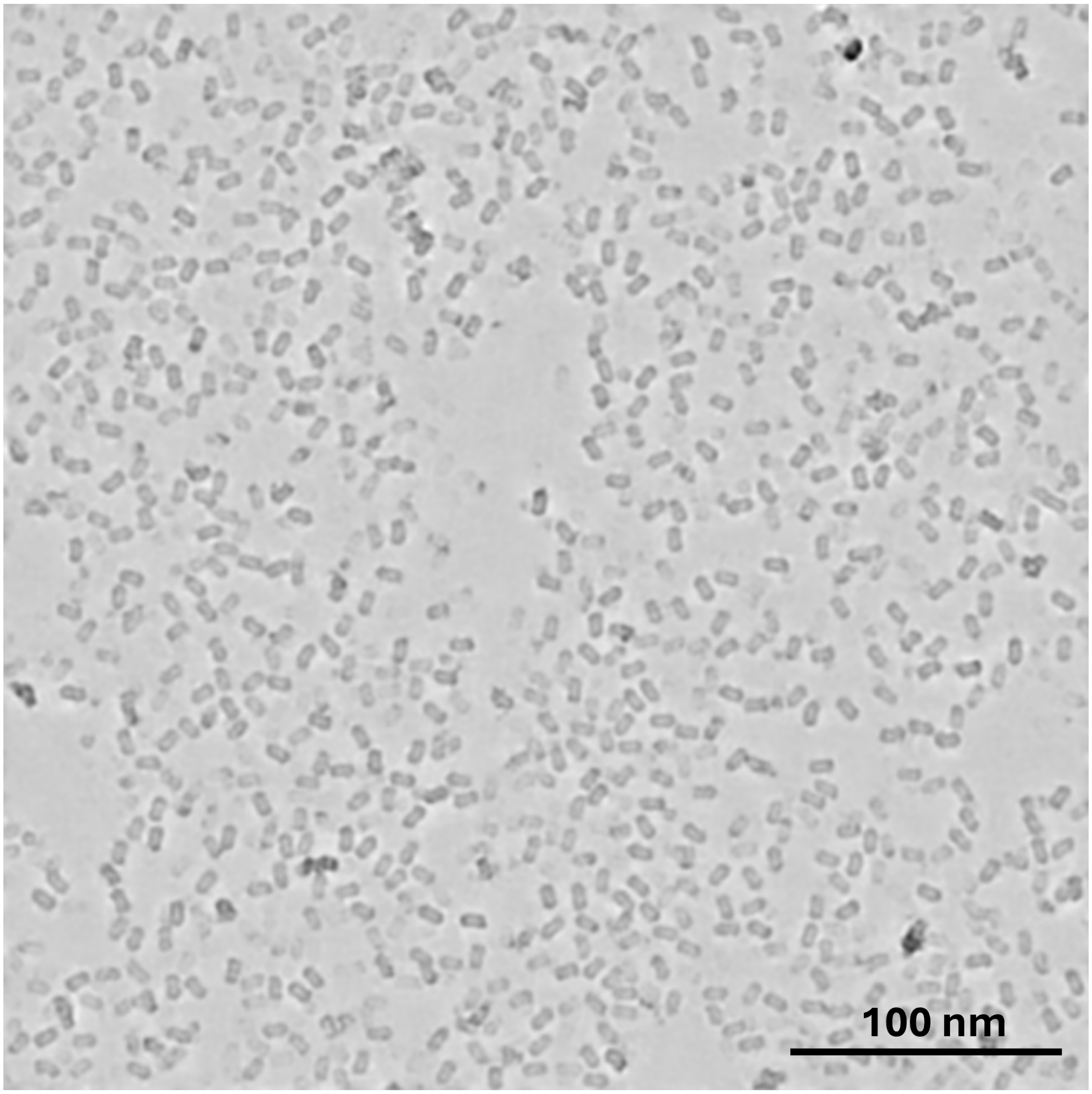

Micrograph was denoised using the cryoSPARC micrograph denoiser. At this protein concentration, *E. coli* TGT predominantly forms dimers that exhibit severe preferred orientation. A minor population of dimer-of-dimer particles was also observed.

Fig. S2. Cryo-EM micrograph of *E. coli* TGT at 145 µM.

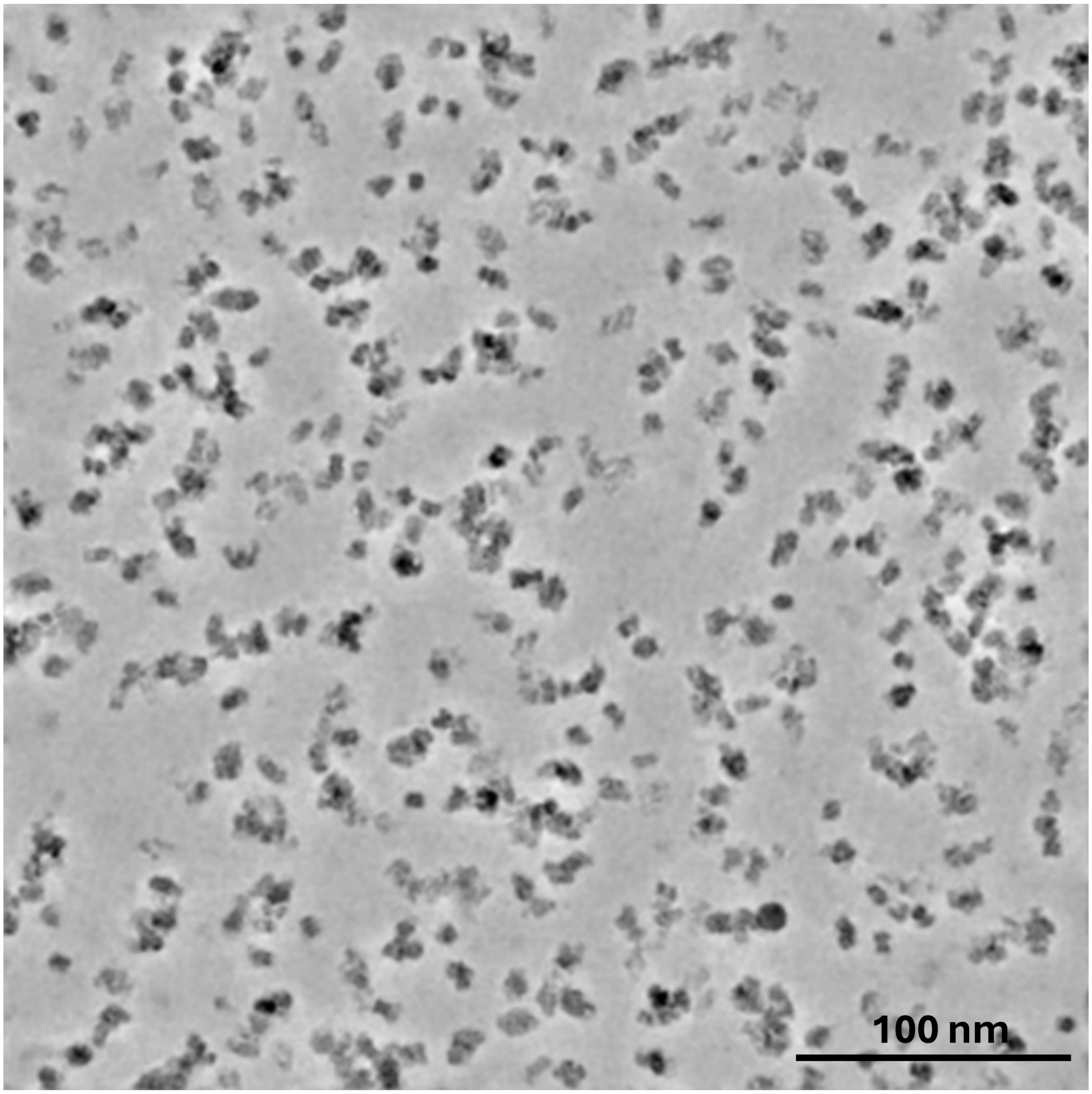

Micrograph was denoised using the cryoSPARC micrograph denoiser. At this protein concentration, *E. coli* TGT predominantly forms a tetramer, with a minor population of higher-order assemblies also present. This sample was used for structure determination of TGT.

Fig. S3. Cryo-EM reconstruction of the TGT-tRNA^{Tyr} covalent intermediate at 2.84 Å resolution (no symmetry imposed)

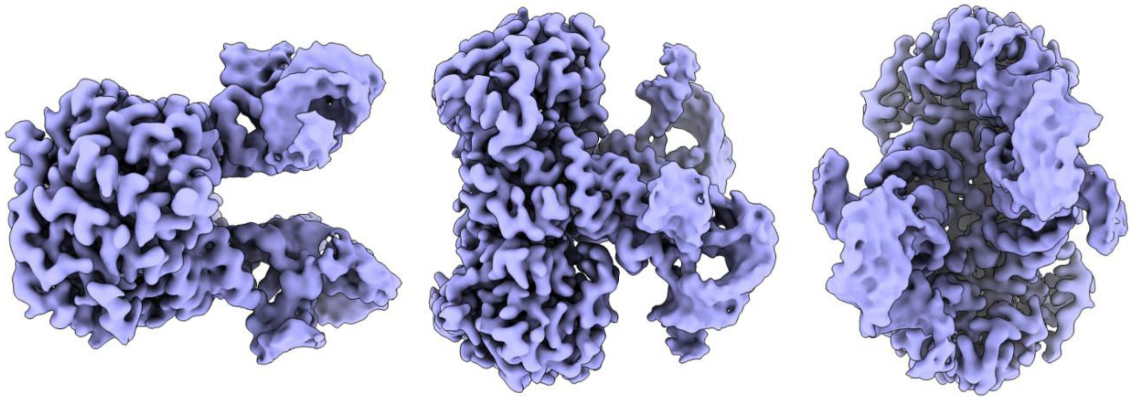

This map was generated using non-uniform refinement without symmetry assigned. The map shows strong density for both tRNAs without assigning C2 symmetry.

Fig. S4. Active site of the TGT–covalent intermediate.

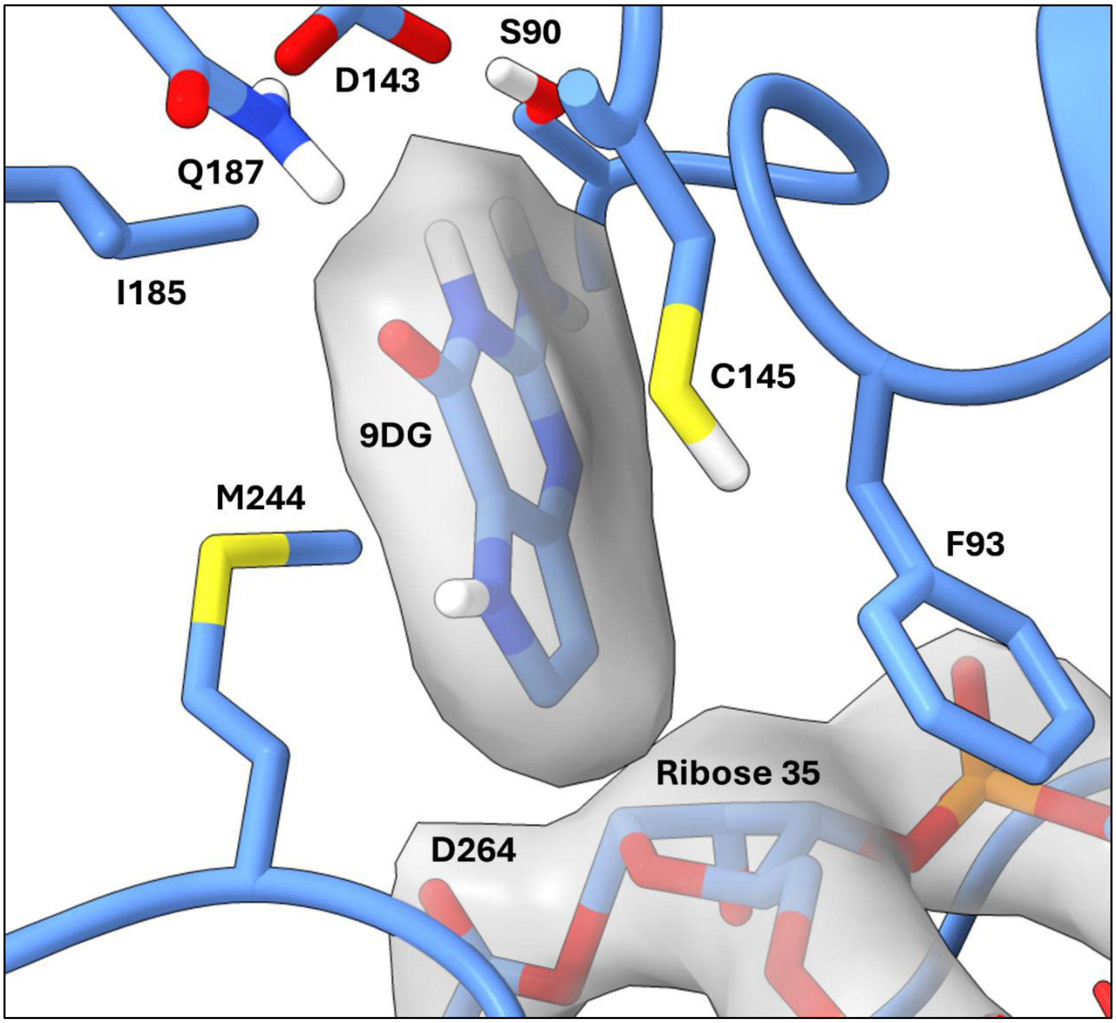

A zoomed-in view of the covalent intermediate with two tRNAs is shown. The EM map shows strong density for the ligand 9-deazaguanine (9DG) in the TGT active site. Additionally, clear density corresponding to the covalent linkage between D264 and ribose 35 of the tRNA is observed. Both the 9DG density and the covalent bond are present in both monomers of the TGT dimer, independent of symmetry assignment during reconstruction.

Fig. S5. Cryo-EM structure of the *E. coli* TGT-tRNA^{Tyr} covalent intermediate with one tRNA.

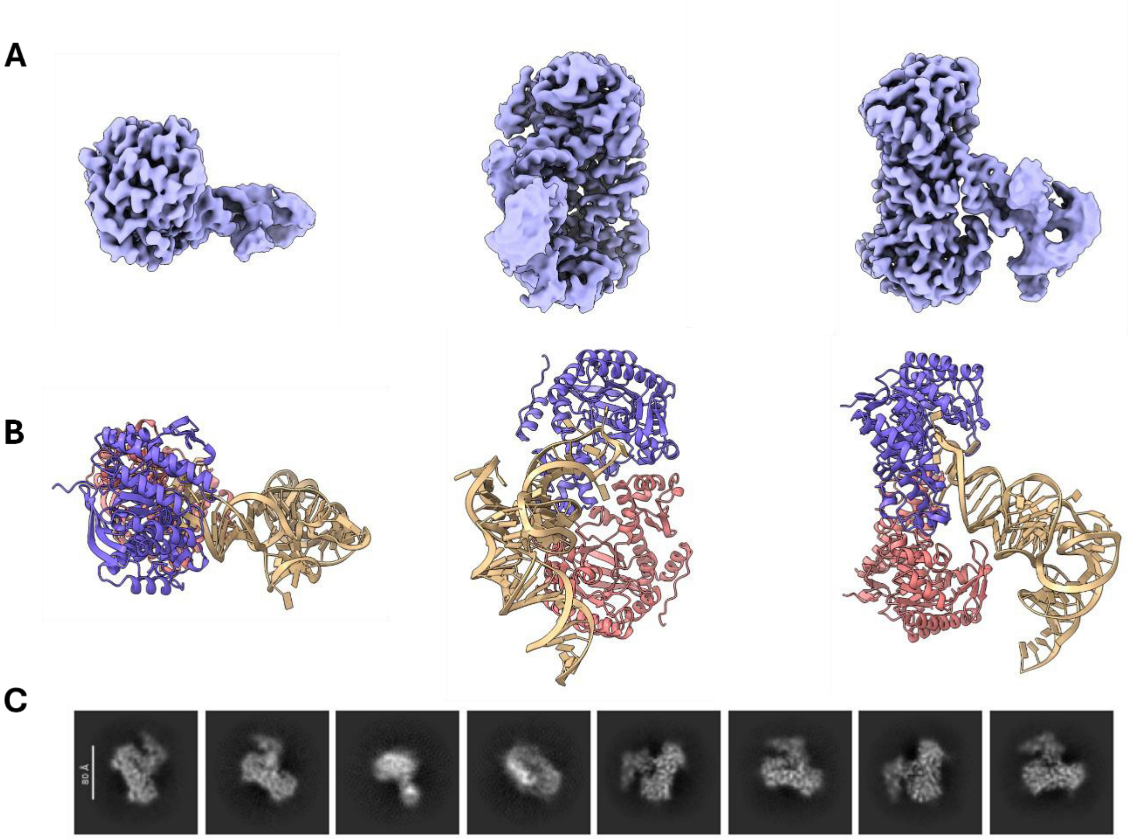

A, Representative views of the 3.04 Å cryo-EM map of the TGT-tRNA^{Tyr} covalent intermediate with only 1 tRNA bound. **B**, Corresponding model of the TGT-tRNA^{Tyr} covalent intermediate built from cryo-EM map. Monomers of TGT in the functional homodimer are colored in blue and red. tRNA^{Tyr} is colored in ochre. **C**, Representative 2D-class averages of the TGT-tRNA^{Tyr} covalent intermediate with only 1 tRNA bound.

Fig. S6. Sequence alignment of *E. coli* and *Z. mobilis* TGT.

```
                0         10         20         30         40         50         60
  E. Coli     MKFEL DTTDG RARRG RLVFD RGVVE TPCFM PVGTY GTVKG MTPEE VEATG AQIIL GNTFH
  Z. Mobilis  FSFSI AAREG KARTG TIEMK RGVIR TPAFM PVGTA ATVKA LKPET VRATG ADIIL GNTYH

                          70         80         90        100        110        120
  E. Coli     LWLRP GQEIM KLHGD LHDFM QWKGP ILTDS GGFQV FSLGD IRKIT EQGVH FRNPI NGDPI
  Z. Mobilis  LMLRP GAERI AKLGG LHSFM GWDRP ILTDS GGYQV MSLSS LTKQS EEGVT FKSHL DGSRH

                         130        140        150        160        170        177
  E. Coli     FLDPE KSMEI QYDLG SDIVM IFDEC TPYPA DWDYA KRSME MSLRW AKRSR ERFDS ---LG
  Z. Mobilis  MLSPE RSIEI QHLLG SDIVM AFDEC TPYPA TPSRA ASSME RSMRW AKRSR DAFDS RKEQA

                         187        197        207        217        227        237
  E. Coli     NKNAL FGIIQ GSVYE DLRDI SVKGL VDIGF DGYAV GGLAV GEPKA DMHRI LEHVC PQIPA
  Z. Mobilis  ENAAL FGIQQ GSVFE NLRQQ SADAL AEIGF DGYAV GGLAV GEGQD EMFRV LDFSV PMLPD

                         247        257        267        277        287        297
  E. Coli     DKPRY LMGVG KPEDL VEGVR RGIDM FDCVM PTRNA RNGHL FVTDG VVKIR NAKYK SDTGP
  Z. Mobilis  DKPHY LMGVG KPDDI VGAVE RGIDM FDCVL PTRSG RNGQA FTWDG PINIR NARFS EDLTP

                         307        317        327        337        347        357
  E. Coli     LDPEC DCYTC RNYSR AYLHH LDRCN EILGA RLNTI HNLRY YQRLM AGLRK AIEEG KLESF
  Z. Mobilis  LDSEC HCAVC QKWSR AYIHH LIRAG EILGA MLMTE HNIAF YQQLM QKIRD SISEG RFSQF

                         367
  E. Coli     VTDFY QRQGR EVPPL NVD
  Z. Mobilis  AQDFR ARYFA RNS-- ---
```

Sequence alignment of *Z. mobilis* and *E. coli* TGT. Residues previously shown to interact with RNA in Z. mobilis TGT[36] that are conserved in *E. coli* TGT are highlighted in blue. RNA-interacting residues in *E. coli* TGT that are mutated in *Z. mobilis* TGT are highlighted in red.

Fig. S7. Comparison of selected RNA-interacting residues of *E. coli* TGT with TGTs from other organisms.

**Residue**	35	112	285	292	320
E. coli	Y	R	K	K	R
Shigella	Y	R	K	K	R
Salmonella	Y	R	K	K	R
V. cholerae	Y	R	K	K	R
Y. pestis	Y	K	K	K	R
Z. mobilis	A	K	N	R	R
H. sapiens	Q	R	Q	V	H

In TGTs from several disease-relevant bacteria (including *Shigella*, *Salmonella*, and *Vibrio cholerae*), most RNA-interacting residues of *E. coli* TGT are conserved. In some bacterial TGTs, one or more of these residues are mutated (e.g., *Y. pestis*), whereas in *Z. mobilis* and human TGT nearly all these residues are mutated.

Fig. S8. Differential scanning fluorimetry (DSF) of single TAG3 and TAG3(n20)TAG3

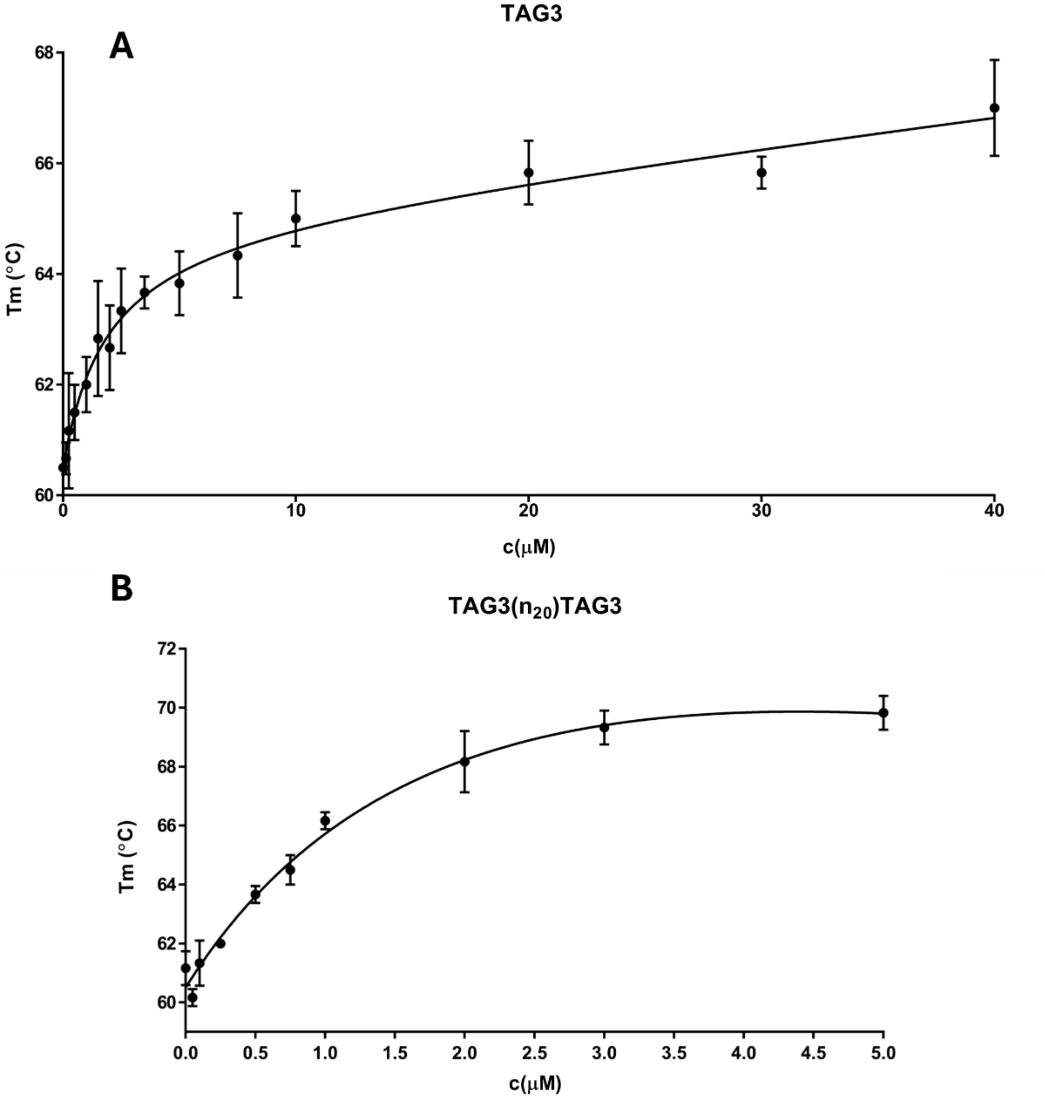

TGT (2.5 μM) was mixed with SYPRO™ Orange (5× final concentration) in TGT reaction buffer (100 mM HEPES pH 7.3, 5 mM DTT, 20 mM $MgCl_2$) on ice. Increasing concentrations of RNA (TAG3 or TAG3(n20)TAG3) were then added. The resulting mixtures were immediately subjected to melt curve analysis using SYPRO™ Orange fluorescence as a readout. Melt curves were generated in triplicate. Melting temperatures (T_m) of TGT were plotted against the respective RNA concentration, and curves were fitted using a single-site binding model. The apparent K_D was inferred from the RNA concentration at the midpoint of the ΔT_m transition.

TAG3 behaves as a weaker binder, with a binding curve that does not reach full saturation even at RNA concentrations >10 μM. Correspondingly, the observed ΔT_m of ~7 °C yields an apparent K_D of ~3–5 μM. In contrast, TAG3(n20)TAG3 shows substantially higher affinity, with the binding curve reaching saturation at approximately 3 μM RNA. Binding results in a larger ΔT_m of ~9 °C, indicating greater thermal stabilization of the RNA-protein complex. With an apparent K_D of 0.7–1 μM it shows higher binding affinity to TGT than single TAG3.

Fig. S9. Cryo-EM data processing workflow for the structure of TGT.

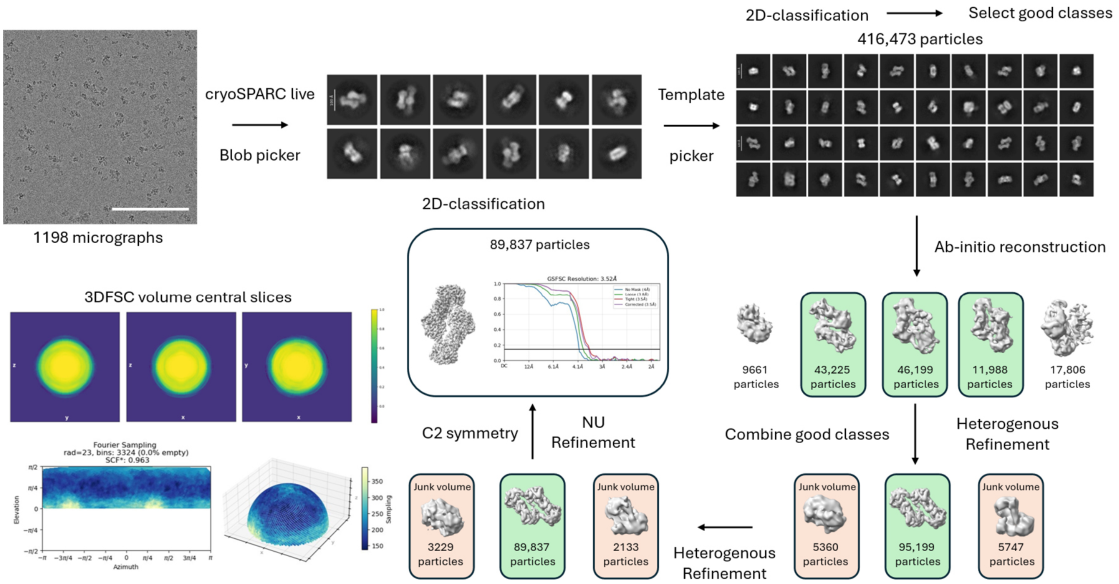

Initial particle picks were obtained in cryoSPARC live. 2D-class averages generated in cryoSPARC live were used for template picking. Good 2D-classes were combined to create 4 ab-initio structures. Particles from good ab-initio structures were combined and further sorted using 2 rounds of heterogeneous refinement. A final round of non-uniform refinement with C2 symmetry assigned was used to generate the final 3.52 Å map. 3DFSC[1,2] and sphericity metric were generated in cryoSPARC.

Fig. S10. Cryo-EM data processing workflow for the TGT-tRNA^{Tyr} covalent intermediate with two bound tRNAs.

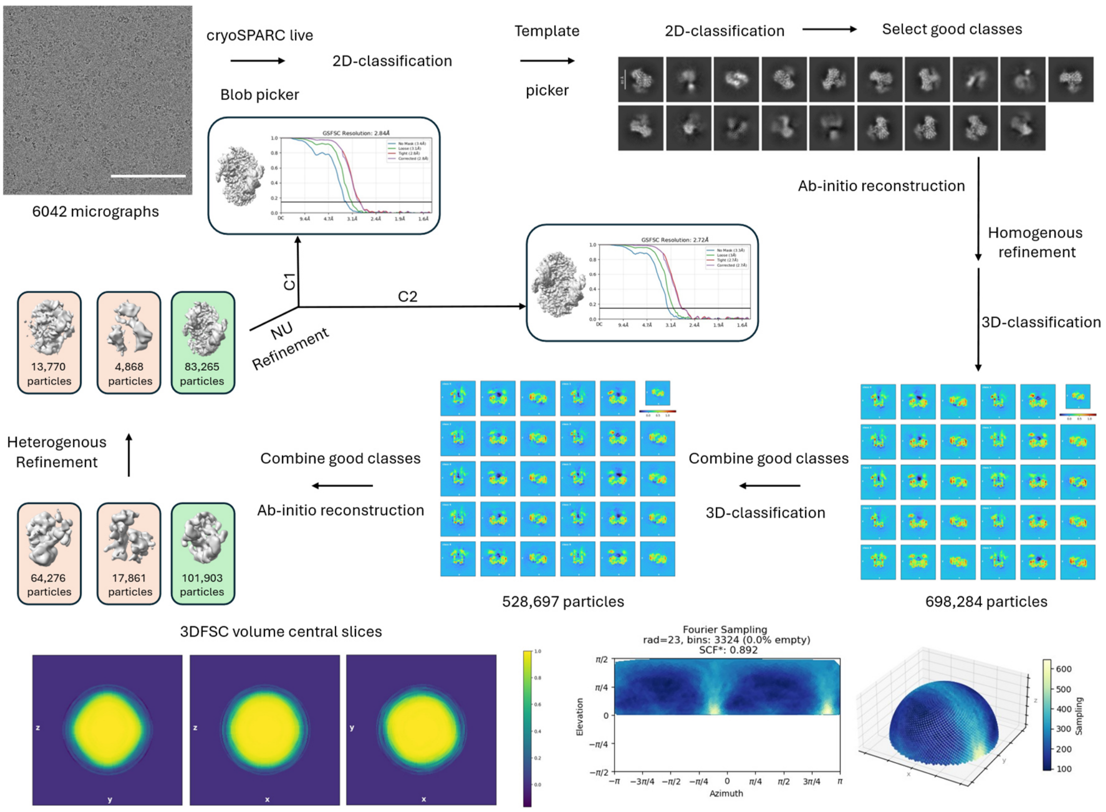

Initial particle picks were obtained in cryoSPARC live. 2D-class averages generated in cryoSPARC live were used for template picking. Good 2D-classes were combined and particles were sorted through 2 rounds of 3D-classification. Good classes were combined and used for generating 3 ab-initio reconstructions refinement. One round of heterogenous refinement then gave the final particle stack. NU-refinement with C1 symmetry gave a 2.84 Å resolution map. Assigning C2 symmetry improved resolution to 2.72 Å. 3DFSC[1,2] and sphericity metric were generated in cryoSPARC.

Fig. S11. Cryo-EM data processing workflow for the TGT-tRNA^{Tyr} covalent intermediate with one bound tRNA.

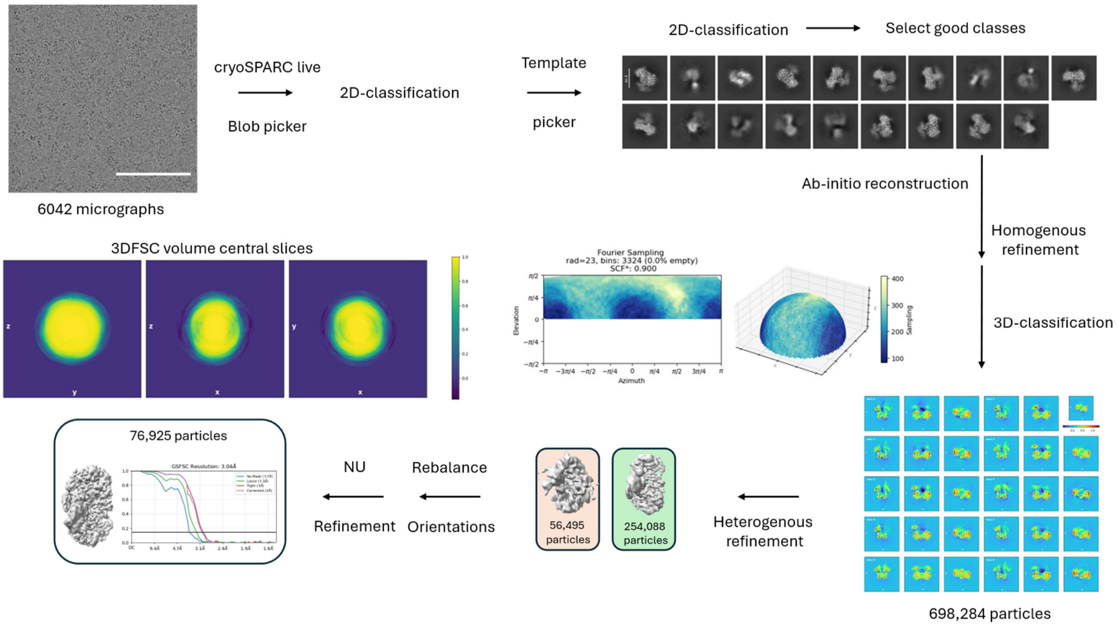

Initial particle picks were obtained in cryoSPARC live. 2D-class averages generated in cryoSPARC live were used for template picking. Good 2D-classes were combined and particles were sorted through one round of 3D-classification. Good classes were combined and further sorted through one round of heterogenous refinement. Orientations had to be rebalanced due to orientation bias in the sample. NU-refinement gave a 3.04 Å resolution map. 3DFSC[1,2] and sphericity metric were generated in cryoSPARC.

***E. coli* TGT expression plasmid used in this study:**

T7-2 *E.coli* TGT:
(Protein encoding sequence is underlined)
AACTACGTCAGGTGGCACTTTTCGGGGAAATGTGCGCGGAACCCCTATTTGTTTATTTTTCTA
AATACATTCAAATATGTATCCGCTCATGAGACAATAACCCTGATAAATGCTTCAATAATATTGA
AAAAGGAAGAGTATGAGTATTCAACATTTCCGTGTCGCCCTTATTCCCTTTTTTGCGGCATTT
TGCCTTCCTGTTTTTGCTCACCCAGAAACGCTGGTGAAAGTAAAAGATGCTGAAGATCAGTT
GGGTGCACGAGTGGGTTACATCGAACTGGATCTCAACAGCGGTAAGATCCTTGAGAGTTTT
CGCCCCGAAGAACGTTTCCCAATGATGAGCACTTTTAAAGTTCTGCTATGTGGCGCGGTATT
ATCCCGTGTTGACGCCGGGCAAGAGCAACTCGGTCGCCGCATACACTATTCTCAGAATGAC
TTGGTTGAGTACTCACCAGTCACAGAAAAGCATCTTACGGATGGCATGACAGTAAGAGAATT
ATGCAGTGCTGCCATAACCATGAGTGATAACACTGCGGCCAACTTACTTCTGACAACGATCG
GAGGACCGAAGGAGCTAACCGCTTTTTTGCACAACATGGGGGATCATGTAACTCGCCTTGAT
CGTTGGGAACCGGAGCTGAATGAAGCCATACCAAACGACGAGCGTGACACCACGATGCCTG
TAGCAATGGCAACAACGTTGCGCAAACTATTAACTGGCGAACTACTTACTCTAGCTTCCCGG
CAACAATTAATAGACTGGATGGAGGCGGATAAAGTTGCAGGACCACTTCTGCGCTCGGCCC
TTCCGGCTGGCTGGTTTATTGCTGATAAATCTGGAGCCGGTGAGCGTGGGTCTCGCGGTAT
CATTGCAGCACTGGGGCCAGATGGTAAGCCCTCCCGTATCGTAGTTATCTACACGACGGGG
AGTCAGGCAACTATGGATGAACGAAATAGACAGATCGCTGAGATAGGTGCCTCACTGATTAA
GCATTGGTAACTGTCAGACCAAGTTTACTCATATATACTTTAGATTGATTTACCCCGGTTGAT
AATCAGAAAAGCCCCAAAAACAGGAAGATTGTATAAGCAAATATTTAAATTGTAAACGTTAAT
ATTTTGTTAAAATTCGCGTTAAATTTTTGTTAAATCAGCTCATTTTTTAACCAATAGGCCGAAA
TCGGCAAAATCCCTTATAAATCAAAAGAATAGCCCGAGATAGGGTTGAGTGTTGTTCCAGTTT
GGAACAAGAGTCCACTATTAAAGAACGTGGACTCCAACGTCAAAGGGCGAAAAACCGTCTAT
CAGGGCGATGGCCCACTACGTGAACCATCACCCAAATCAAGTTTTTTGGGGTCGAGGTGCC
GTAAAGCACTAAATCGGAACCCTAAAGGGAGCCCCCGATTTAGAGCTTGACGGGGAAAGCC
GGCGAACGTGGCGAGAAAGGAAGGGAAGAAAGCGAAAGGAGCGGGCGCTAGGGCGCTGG
CAAGTGTAGCGGTCACGCTGCGCGTAACCACCACACCCGCCGCGCTTAATGCGCCGCTACA
GGGCGCGTAAAAGGATCTAGGTGAAGATCCTTTTTGATAATCTCATGACCAAAATCCCTTAA
CGTGAGTTTTCGTTCCACTGAGCGTCAGACCCCGTAGAAAAGATCAAAGGATCTTCTTGAGA
TCCTTTTTTTCTGCGCGTAATCTGCTGCTTGCAAACAAAAAAACCACCGCTACCAGCGGTGG
TTTGTTTGCCGGATCAAGAGCTACCAACTCTTTTTCCGAAGGTAACTGGCTTCAGCAGAGCG
CAGATACCAAATACTGTCCTTCTAGTGTAGCCGTAGTTAGGCCACCACTTCAAGAACTCTGT
AGCACCGCCTACATACCTCGCTCTGCTAATCCTGTTACCAGTGGCTGCTGCCAGTGGCGAT
AAGTCGTGTCTTACCGGGTTGGACTCAAGACGATAGTTACCGGATAAGGCGCAGCGGTCGG
GCTGAACGGGGGGTTCGTGCACACAGCCCAGCTTGGAGCGAACGACCTACACCGAACTGA
GATACCTACAGCGTGAGCTATGAGAAAGCGCCACGCTTCCCGAAGGGAGAAAGGCGGACA
GGTATCCGGTAAGCGGCAGGGTCGGAACAGGAGAGCGCACGAGGGAGCTTCCAGGGGGA
AACGCCTGGTATCTTTATAGTCCTGTCGGGTTTCGCCACCTCTGACTTGAGCGTCGATTTTT
GTGATGCTCGTCAGGGGGGCGGAGCCTATGGAAAAACGCCAGCAACGCGGCCTTTTTACG
GTTCCTGGCCTTTTGCTGGCCTTTTGCTCACATGTTCTTTCCTGCGTTATCCCCTGATTCTGT
GGATAACCGTATTACCGCCTTTGAGTGAGCTGATACCGCTCGCCGCAGCCGAACGACCGAG
CGCAGCGAGTCAGTGAGCGAGGAAGCTATGGTGCACTCTCAGTACAATCTGCTCTGATGCC
GCATAGTTAAGCCAGTATACACTCCGCTATCGCTACGTGACTGGGTCATGGCTGCGCCCCG
ACACCCGCCAACACCCGCTGACGCGCCCTGACGGGCTTGTCTGCTCCCGGCATCCGCTTA
CAGACAAGCTGTGACCGTCTCCGGGAGCTGCATGTGTCAGAGGTTTTCACCGTCATCACCG
AAACGCGCGAGGCAGCTGCGGTAAAGCTCATCAGCGTGGTCGTGCAGCGATTCACAGATGT
CTGCCTGTTCATCCGCGTCCAGCTCGTTGAGTTTCTCCAGAAGCGTTAATGTCTGGCTTCTG
ATAAAGCGGGCCATGTTAAGGGCGGTTTTTTCCTGTTTGGTCACTGATGCCTCCGTGTAAGG
GGGATTTCTGTTCATGGGGGTAATGATACCGATGAAACGAGAGAGGATGCTCACGATACGG
GTTACTGATGATGAACATGCCCGGTTACTGGAACGTTGTGAGGGTAAACAACTGGCGGTAT
GGATGCGGCGGGACCAGAGAAAAATCACTCAGGGTCAATGCCAGCCGAACGCCAGCAAGA
CGTAGCCCAGCGCGTCGGCCGCCATGCCGGCGATAATGGCCTGCTTCTCGCCGAAACGTT
TGGTGGCGGGACCAGTGACGAAGGCTTGAGCGAGGGCGTGCAAGATTCCGAATACCGCAA
GCGACAGGCCGATCATCGTCGCGCTCCAGCGAAAGCGGTCCTCGCCGAAAATGACCCAGA

GCGCTGCCGGCACCTGTCCTACGAGTTGCATGATAAAGAAGACAGTCATAAGTGCGGCGAC
GATAGTCATGCCCCGCGCCCACCGGAAGGAGCTGACTGGGTTGAAGGCTCTCAAGGGCAT
CGGTCGAGATCCCGGTGCCTAATGAGTGAGCTAACTTACATTAATTGCGTTGCGCTCACTGC
CCGCTTTCCAGTCGGGAAACCTGTCGTGCCAGCTGCATTAATGAATCGGCCAACGCGCGGG
GAGAGGCGGTTTGCGTATTGGGCGCCAGGGTGGTTTTTCTTTTCACCAGTGAGACGGGCAA
CAGCTGATTGCCCTTCACCGCCTGGCCCTGAGAGAGTTGCAGCAAGCGGTCCACGCTGGTT
TGCCCCAGCAGGCGAAAATCCTGTTTGATGGTGGTTAACGGCGGGATATAACATGAGCTGT
CTTCGGTATCGTCGTATCCCACTACCGAGATGTCCGCACCAACGCGCAGCCCGGACTCGGT
AATGGCGCGCATTGCGCCCAGCGCCATCTGATCGTTGGCAACCAGCATCGCAGTGGGAAC
GATGCCCTCATTCAGCATTTGCATGGTTTGTTGAAAACCGGACATGGCACTCCAGTCGCCTT
CCCGTTCCGCTATCGGCTGAATTTGATTGCGAGTGAGATATTTATGCCAGCCAGCCAGACGC
AGACGCGCCGAGACAGAACTTAATGGGCCCGCTAACAGCGCGATTTGCTGGTGACCCAATG
CGACCAGATGCTCCACGCCCAGTCGCGTACCGTCTTCATGGGAGAAAATAATACTGTTGATG
GGTGTCTGGTCAGAGACATCAAGAAATAACGCCGGAACATTAGTGCAGGCAGCTTCCACAG
CAATGGCATCCTGGTCATCCAGCGGATAGTTAATGATCAGCCCACTGACGCGTTGCGCGAG
AAGATTGTGCACCGCCGCTTTACAGGCTTCGACGCCGCTTCGTTCTACCATCGACACCACCA
CGCTGGCACCCAGTTGATCGGCGCGAGATTTAATCGCCGCGACAATTTGCGACGGCGCGT
GCAGGGCCAGACTGGAGGTGGCAACGCCAATCAGCAACGACTGTTTGCCCGCCAGTTGTT
GTGCCACGCGGTTGGGAATGTAATTCAGCTCCGCCATCGCCGCTTCCACTTTTTCCCGCGTT
TTCGCAGAAACGTGGCTGGCCTGGTTCACCACGCGGGAAACGGTCTGATAAGAGACACCG
GCATACTCTGCGACATCGTATAACGTTACTGGTTTCACATTCACCACCCTGAATTGACTCTCT
TCCGGGCGCTATCATGCCATACCGCGAAAGGTTTTGCGCCATTCGATGGTGTCCGGGATCT
CGACGCTCTCCCTTATGCGACTCCTGCATTAGGAAGCAGCCCAGTAGTAGGTTGAGGCCGT
TGAGCACCGCCGCCGCAAGGAATGGTGCATGCCGGCATGCCGCCCTTTCGTCTTCAAGAAT
TAATTCCCAATTCCCCAGGCATCAAATAAAACGAAAGGCTCAGTCGAAAGACTGGGCCTTTC
GTTTTATCTGTTGTTTGTCGGTGAACGCTCTCCTGAGTAGGACAAATCCGCCGGGAGCGGAT
TTGAACGTTGCGAAGCAACGGCCCGGAGGGTGGCGGGCAGGACGCCCGCCATAAACTGCC
AGGAATTAATTCCCCAGGCATCAAATAAAACGAAAGGCTCAGTCGAAAGACTGGGCCTTTCG
TTTTATCTGTTGTTTGTCGGTGAACGCTCTCCTGAGTAGGACAAATCCGCCGGGAGCGGATT
TGAACGTTGCGAAGCAACGGCCCGGAGGGTGGCGGGCAGGACGCCCGCCATAAACTGCCA
GGAATTAATTCCCCAGGCATCAAATAAAACGAAAGGCTCAGTCGAAAGACTGGGCCTTTCGT
TTTATCTGTTGTTTGTCGGTGAACGCTCTCCTGAGTAGGACAAATCCGCCGGGAGCGGATTT
GAACGTTGCGAAGCAACGGCCCGGAGGGTGGCGGGCAGGACGCCCGCCATAAACTGCCA
GGAATTAATTCCCCAGGCATCAAATAAAACGAAAGGCTCAGTCGAAAGACTGGGCCTTTCGT
TTTATCTGTTGTTTGTCGGTGAACGCTCTCCTGAGTAGGACAAATCCGCCGGGAGCGGATTT
GAACGTTGCGAAGCAACGGCCCGGAGGGTGGCGGGCAGGACGCCCGCCATAAACTGCCA
GGAATTAATTCCCCAGGCATCAAATAAAACGAAAGGCTCAGTCGAAAGACTGGGCCTTTCGT
TTTATCTGTTGTTTGTCGGTGAACGCTCTCCTGAGTAGGACAAATCCGCCGGGAGCGGATTT
GAACGTTGCGAAGCAACGGCCCGGAGGGTGGCGGGCAGGACGCCCGCCATAAACTGCCA
GGAATTGGGGATCGGAATTAATTCCCGGTTTAAACCGGGGATCTCGATCCCGCGAAATTAAT
ACGACTCACTATAGGGGAATTGTGAGCGGATAACAATTCCCCTCTAGAAATAATTTTGTTTAA
CTTTAAGAAGGAGATATACAATGTGGAGCCATCCGCAGTTTGAAAAAGGCAAATTTGAACTG
GACACCACCGACGGTCGCGCACGCCGTGGCCGCCTGGTCTTTGATCGTGGCGTAGTGGAA
ACGCCTTGTTTTATGCCTGTTGGCACCTACGGCACCGTAAAAGGGATGACGCCGGAAGAAG
TTGAAGCCACTGGCGCGCAAATTATCCTCGGCAACACCTTCCACCTGTGGCTGCGCCCGGG
CCAGGAAATCATGAAACTGCACGGCGATCTGCACGATTTTATGCAGTGGAAGGGGCCGATC
CTCACCGACTCCGGCGGCTTCCAGGTCTTCAGCCTTGGCGATATTCGTAAAATCACCGAACA
GGGCGTGCACTTCCGTAACCCGATCAACGGCGATCCGATTTTCCTCGATCCTGAAAAATCAA
TGGAGATTCAGTACGATCTTGGTTCGGATATCGTCATGATCTTTGATGAGTGTACGCCGTAT
CCTGCTGACTGGGATTACGCAAAACGCTCCATGGAGATGTCTCTGCGTTGGGCGAAGCGTA
GCCGTGAGCGTTTTGACAGTCTCGGAAACAAAAATGCGCTGTTTGGTATCATCCAGGGCAG
CGTTTACGAAGATTTACGTGATATTTCTGTTAAAGGTCTGGTAGATATCGGTTTTGATGGCTA
CGCTGTCGGCGGTCTGGCTGTGGGTGAGCCGAAAGCAGATATGCACCGCATTCTGGAGCA
TGTATGCCCGCAAATTCCGGCAGACAAACCGCGTTACCTGATGGGCGTTGGTAAACCAGAA
GACCTGGTTGAAGGCGTACGTCGTGGTATCGATATGTTTGACTGCGTAATGCCAACCCGCA
ACGCCCGAAATGGTCATTTGTTCGTGACCGATGGCGTGGTGAAAATCCGCAATGCGAAGTA

TAAGAGCGATACTGGCCCACTCGATCCTGAGTGTGATTGCTACACCTGTCGCAATTATTCAC
GCGCTTACTTGCATCATCTTGACCGTTGCAACGAAATATTAGGCGCGCGACTCAACACCATT
CATAACCTTCGTTACTACCAGCGTTTGATGGCGGGTTTACGCAAGGCTATTGAAGAGGGTAA
ATTAGAGAGCTTCGTAACTGATTTTTACCAGCGTCAGGGGCGAGAAGTACCACCTTTGAACG
TTGATCACCATCACCACCATCACTAAGCGGCCGCATTGATCCGGCTGCTAACAAAGCCCGAA
AGGAAGCTGAGTTGGCTGCTGCCACCGCTGAGCAATAACTAGCATAACCCCTTGGGGCCTC
TAAACGGGTCTTGAGGGGTTTTTTGCTGAAAGGAGGAACTATATCCGGAT

Tables

Table S1: Primers for site-directed mutagenesis:

Primer	Sequence
K285D-fwd	TGGCGTGGTGGATATCCGCAATG
K285D-rev	TCGGTCACGAACAAATGAC
K292D-fwd	TGCGAAGTATGATAGCGATACTGGC
K292D-rev	TTGCGGATTTTCACCAC
R320D-fwd	TCATCTTGACGATTGCAACGAAATATTAGGCG
R320D-rev	TGCAAGTAAGCGCGTG
Y35F-fwd	TGTTGGCACCTTTGGCACCGTAA
Y35F-rev	GGCATAAAACAAGGCGTTTC
R112E-fwd	CGTGCACTTCGAAAACCCGATCAAC
R112E-rev	CCCTGTTCGGTGATTTTAC

Table S2: Cryo-EM Data Collection, refinement, and validation statistics.

	E. coli TGT	*E. coli* TGT covalent intermediate with 1 tRNA	*E. coli* TGT covalent intermediate with 2 tRNAs
**Data collection and processing**			
Microscope	Talos Arctica	Titan Krios G4	Titan Krios G4
Voltage (keV)	200	300	300
Detector	Falcon4	Falcon4	Falcon4
Nominal magnification	150,000	165,000	165,000
Data Acquisition Software	EPU2	EPU2	EPU2
Electron dose (e^{-}/Å^2)	55	55	55
Pixel Size (Å)	0.95	0.735	0.735
Defocus range (µm)	−2.4 to −1.0	−2.2 to −0.6	−2.2 to −0.6
Number of movies (#)	1198	6042	6042
Number of particles	89,837	76,925	83,087
Symmetry imposed	C2	-	C2
Resolution (Å)	3.52	3.04	2.72
FSC threshold	0.143	0.143	0.143
**Refinement**			
Initial model used	Alphafold	Alphafold	Alphafold
Non-hydrogen atoms	11924	13671	15488
Protein residues	1492	750	750
Nucleotides	-	85	170
Zn^{2+}	4	2	2
9DG	-	1	2
R.M.S deviations			
Bond lengths (Å)	0.002	0.003	0.004
Bond angles (°)	0.533	0.610	0.582
**Validation**			
MolProbity score	1.51	1.56	1.64
Clashscore	6.51	5.49	6.47
Poor rotamers (%)	1.25	0.16	1.09
Ramachandran (%)			
Favored	97.63%	96.11%	96.25%
Allowed	2.37%	3.89%	3.75%
Disallowed	0.00 %	0.00 %	0.00 %
Fit to map (CC_{mask})	0.80	0.82	0.89
**Accession codes**			
EMDB	EMD-75124	EMD-75125	EMD-75126
PDB	10FA	10FB	10FC

SI References

[1] P. R. Baldwin, D. Lyumkis, Non-Uniformity of Projection Distributions Attenuates Resolution in Cryo-EM. *Prog. Biophys. Mol. Biol.* **150**, 160-183 (2020).
[2] Y. Tan, et al., Addressing preferred specimen orientation in single-particle cryo-EM through tilting. *Nat. Methods.* **14**, 793-796 (2017).
